# A method for validating the accuracy of NMR protein structures

**DOI:** 10.1038/s41467-020-20177-1

**Published:** 2020-12-18

**Authors:** Nicholas J. Fowler, Adnan Sljoka, Mike P. Williamson

**Affiliations:** 1 Dept of Molecular Biology and Biotechnology, University of Sheffield, Sheffield, UK; 2 RIKEN Center for Advanced Intelligence Project, RIKEN, 1-4-1 Nihombashi, Chuo-ku, Tokyo 103-0027 Japan; 3 Dept of Chemistry, University of Toronto, UTM, 3359 Mississauga Road North, Mississauga, ON L5L 1C6 Canada

**Keywords:** Structural biology, Computational biophysics, Solution-state NMR

## Abstract

We present a method that measures the accuracy of NMR protein structures. It compares random coil index [RCI] against local rigidity predicted by mathematical rigidity theory, calculated from NMR structures [FIRST], using a correlation score (which assesses secondary structure), and an RMSD score (which measures overall rigidity). We test its performance using: structures refined in explicit solvent, which are much better than unrefined structures; decoy structures generated for 89 NMR structures; and conventional predictors of accuracy such as number of restraints per residue, restraint violations, energy of structure, ensemble RMSD, Ramachandran distribution, and clashscore. Restraint violations and RMSD are poor measures of accuracy. Comparisons of NMR to crystal structures show that secondary structure is equally accurate, but crystal structures are typically too rigid in loops, whereas NMR structures are typically too floppy overall. We show that the method is a useful addition to existing measures of accuracy.

## Abstract (web summary)

The authors present a method for calculating the accuracy of an NMR structure, where flexibility from backbone chemical shifts is compared to structural flexibility predicted using rigidity theory. The authors validate their method and use it to compare the accuracy of NMR and X-ray structures.

## Introduction

Protein structures are probably the single most important resource for understanding protein function, and are deposited in the protein data bank (PDB), which currently contains around 160,000 structures, of which around 90% are X-ray diffraction structures, 8% are nuclear magnetic resonance (NMR) structures, and the rest are mainly from electron microscopy (EM)^1^. The NMR structures are relatively small in number, but are important because they include a high proportion of small proteins with under-represented folds. Most NMR structures are determined in solution, whereas X-ray structures are determined in a crystalline environment. Arguably this makes NMR structures more representative of in vivo structures. However, structures are only useful if they are accurate (i.e., close to the “true” structure) and (equally importantly) can be shown to be accurate. The PDB has therefore become increasingly concerned about validation of structures in the database: the community needs objective and reliable measures to check whether the structure deposited is accurate. The PDB set up four task forces to provide recommendations for validation: for crystallography, NMR, EM, and small-angle scattering, which have all reported^2–5^ and have created a suite of validation tools for the PDB^6^. They concluded that validation cannot be based on a single measure. The measures used comprise a combination of geometrical tests, and comparison to input data. Because it is expected that crystal structures and solution structures have the same physical forces underlying them, the geometrical tests for crystal and NMR structures are identical, and include clashscore (how well atoms are packed together), an analysis of Ramachandran outliers (how well the backbone dihedral angles comply with structural norms), and an analysis of sidechain outliers. The comparisons to input data are necessarily different for X-ray and NMR structures. For X-ray structures there is a very good measure, namely the *R* factor, which is the difference between the intensities of experimental diffraction data, and those calculated from the final structure. If the *R* factor is low (typically less than about 20%) then the structure is almost certainly essentially correct. In structural biology there is a strong temptation to over-fit the data, i.e., to add extra detail in order to improve the fit between experimental data and structure. Hence, a second measure was developed: *R*_free_, which is an *R* factor calculated using 10% of the diffraction data that was set aside and not used in the refinement^7^. *R*_free_ should be similar in size to *R* for a structure that is not over-refined. Together these two measures provide a reliable guide to the accuracy of the crystal structure.

Unfortunately, no such measure exists for NMR structures^8–11^. The original experimental data have no direct mathematical relationship with the structure in the way that diffraction data do; and the experimental input restraints, of which the most common and useful are distance restraints obtained from NOESY spectra, require extensive manipulation and interpretation of the original data before they can be used as restraints. Furthermore, the quantity of information comprising the experimental restraints is far less for NMR, and the information is much more local. This makes NMR structures inherently less precise, and probably less accurate too, and also means that cross-validation by missing out 10% of the data, as used for *R*_free_, is not generally possible for NMR structures^12^. NMR structures thus tend to be validated using an unsatisfactory set of restraint comparisons, typically comprising number of restraints per residue, restraint violations, and structure precision (RMS distance between members of the ensemble)^5,13^. None of these is a direct comparison to the input data, and the third of these is explicitly a measure of precision, not of accuracy, and it is already well established that there is little relationship between precision and accuracy^14–17^.

Hence there is a pressing need to find a better validation measure for NMR structures. Here, we present such a measure. A good validation method should (like the *R* factor) as far as possible compare input data directly to structure. The most obvious input data for NMR structures is the spectra. There have been attempts to do this^18,19^ but there are major difficulties: there is no good way of accurately calculating chemical shifts from structures; dynamics in solution have big effects on spectra; there are many experimental artifacts in NMR spectra; and the number and variety of input spectra used in structure calculations makes it hard to define or measure what should be compared. Hence, we have here used backbone chemical shifts as our input data. These can usually be obtained reliably and rapidly, and there is little or no manipulation or sorting required, by contrast to distance restraints. The method described here is named ANSURR (Accuracy of NMR Structures using Random Coil Index and Rigidity).

The structure of this paper is that we outline the method before demonstrating how we have validated the method using a range of “good” and “bad” structures and by comparing to other typical measures of structure accuracy. We then demonstrate the power of the method by using it to make comparisons between crystal structures and NMR structures.

## Results

### Outline of the method

Backbone chemical shift assignments (i.e., HN, ^15^N, ^13^Cα, ^13^Cβ, Hα, and C′) can usually be obtained rapidly, semi-automatically, and reliably from a set of triple resonance spectra obtained from ^15^N, ^13^C double labeled protein. In order to determine a protein NMR structure, shift assignments are the necessary first stage^20^, meaning that any protein that has an NMR structure must have backbone shift assignments (which are now required to be submitted with the structures). Crucially, shift assignments are subject to minimal manipulation. This is very different from distance restraints obtained from NOE spectra. For distance restraints there are inevitably many stages of data sorting and rejection, no matter whether the restraints are inputted manually or automatically. Some person or computer must decide which signals to include, how to assign them, when to reject or modify the restraints, and how to set the calibration between peak intensity and distance restraint. All of these reduce the value of distance restraints as independent quality measures. For all these reasons, backbone assignments are better validation input than distance restraints.

In our method, backbone chemical shift assignments are compared to a structure. Although a number of programs can calculate shifts from structures, they are not sufficiently accurate to perform a useful comparison except in rather general terms^14,21^. Hence, the heart of our method is that the backbone shifts are used to calculate the local rigidity of the backbone, based on an established measure, the random coil index (RCI), which calculates how similar each of the six backbone shifts is to a tabulated “random coil shift” value^22^. It has been shown to provide a remarkably reliable guide to local rigidity, whether measured by NMR relaxation or by crystallographic *B* factor^22,23^.

We compare local rigidity as predicted by RCI to that computed from a structure using techniques from mathematical rigidity theory. Several software packages and methodologies relying on rigidity theory such as the program Floppy Inclusions and Rigid Substructure Topography (FIRST)^24,25^ and its various implementations and extensions have been developed for fast computational predictions of rigidity and flexibility of protein structures. Starting with a protein structure, FIRST creates a topological graph (a constrained network consisting of nodes and edges), where atoms are represented by vertices (nodes), and edges represent the constraints corresponding to the intramolecular interactions of a protein e.g., covalent bonds, hydrogen bonds and hydrophobic interactions. Applying the mathematically well-established pebble game algorithm and molecular theorem^26^, FIRST then determines locally rigid subgraphs (rigid regions in the network), a process referred to as rigid cluster decomposition. The degree of flexibility can be quantified as a function of hydrogen bond energy by repeating rigid cluster decomposition as edges corresponding to hydrogen bonds are removed incrementally from the graph, and noting the energy at which the Cα atom of a residue no longer belongs to a rigid subgraph, i.e., becomes flexible. We convert this energy to a Boltzmann population ratio, effectively giving the probability that a residue is flexible.

The two measures of local rigidity (RCI and FIRST) are then compared and a numerical comparison gives a score: a measure of how well the local rigidities match, and thus whether the structures produce a local rigidity that matches the one described by the RCI. Following extensive trials, we use two different measures of similarity: (a) The *correlation* between the two. This tests whether the peaks and troughs are in the same places. Peaks are locally mobile regions while troughs are locally rigid regions, generally regular secondary structure. This comparison therefore mainly shows whether the secondary structure is correct. (b) The *root-mean square deviation* (RMSD) between the two. This tests whether overall the structure is too rigid or too floppy. It is strongly influenced by the geometry of hydrogen bonds and other non-covalent interactions in the structure. As discussed below, the overall rigidity of a structure is determined by not just backbone but also sidechain interactions. Protein structures are often compared by superimposing backbones (often cartoons). Two structures can look very similar in a comparison like this, but one can be much worse than the other in terms of the accuracy of the hydrogen bond network or side chain orientations. In order to assess the relationship between structure and function, it is important that sidechain positions should be correct. The RMSD measure between RCI and FIRST is therefore important because it measures the kind of accuracy needed to interpret function.

Correlation and RMSD are simple numerical values, but they do not scale linearly to intuitive measures of accuracy. In the output from ANSURR, we therefore present the numerical values, but we also calculate the percentile of each measure relative to all NMR structures in the PDB with good chemical shift completeness (see below for further discussion of completeness), which we term *correlation score* and *RMSD score*, respectively. These are relative values (and are thus likely to change slightly as more structures are added to the PDB), but are easier for the user to interpret. The crystallographic validations in the PDB adopt a similar procedure for both geometrical tests and *R*_free_. In what follows, we report the scores rather than the numerical values.

**Fig. 1:**
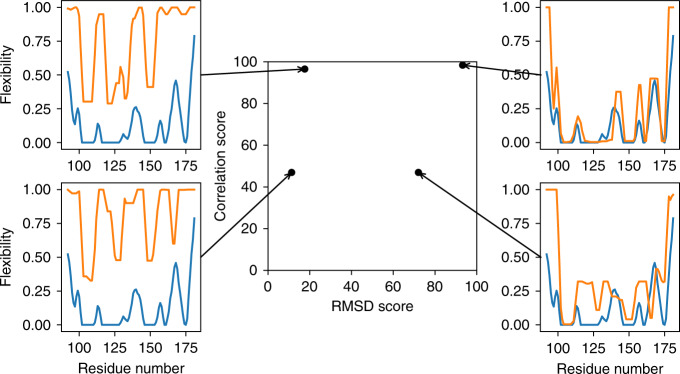
ANSURR analysis of four models from NMR ensembles for the DNA binding domain of the human Forkhead transcription factor AFX (PDB ID 1e17). In the four plots, the blue lines show the flexibility predicted by RCI while the orange lines show flexibility predicted by FIRST. In the center of the figure is the ANSURR analysis showing the RMSD and correlation scores derived from the four models. The two models on the right are from the CNW dataset^27^ (refined in explicit solvent), while the two on the left are from the CNS set (refined in vacuo). As is typical, the CNW-refined structures have better RMSD, meaning that the calculated flexibilities compare well on average. The two models at the bottom have poor correlations, because the locations of the peaks do not match well between RCI and FIRST. The two at the top both have good correlations, because the locations of the peaks do match, even though (in the case of the top left structure) their heights are very different.

Correlation and RMSD scores highlight different aspects of accuracy, so we decided not to combine them into a single score to represent overall accuracy. Instead, we plot both on a single graph, as demonstrated in Fig. 1 for four different models of the same protein. The most accurate models (those with good scores for both correlation and RMSD) appear in the top right-hand corner of the plot.

### RECOORD CNS (unrefined) vs. CNW (refined) structures

There is currently no accepted method for measuring the accuracy of an NMR structure. There are also no databases of “good” or “bad” structures. We have therefore created or adopted datasets that can reasonably be assumed to be bad or good. There are also a range of methods that have been used to measure structure quality, including the geometrical methods described above. We compare our findings to these methods in turn.

The RECOORD project^27^ set out to standardize and tabulate methods for NMR structure calculation. It produced a curated set of structure restraints, which were applied in a consistent manner to more than 500 proteins from the PDB, and then analysed the resultant structures. It carried out two sets of structure calculations on each protein: one using a typical simulated annealing calculation in vacuo using CNS (termed CNS) and another using CYANA (termed CYA)^28,29^. They then took these two sets of structures and refined them in explicit water using ARIA (termed CNW and CYW, respectively)^30^. There is an extensive literature indicating that refinement of NMR structures in explicit water produces better geometries and generally better quality structures^31^, so not surprisingly, the CNW/CYW structures are better.

**Fig. 2:**
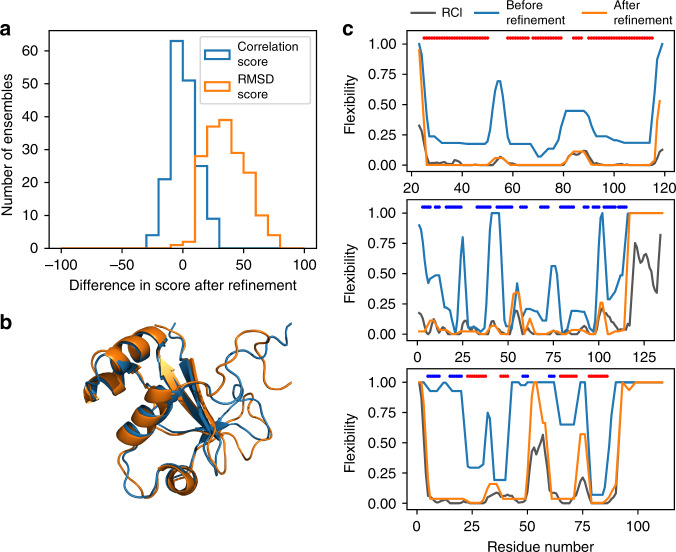
The effect of explicit solvent refinement on the two measures of structure accuracy. **a** Histogram showing the change in average correlation score (blue) and RMSD score (orange), comparing ensembles from the CNS75 to the CNW75 sets. RMSD scores improve dramatically while there is no significant change in correlation scores. **b** Backbone superposition of CNS model 14 and CNW model 14 of the restriction of telomere capping protein 3 from *S. cerevisiae* (PDB ID 1nyn), as a typical example of the effect of refinement in explicit solvent. Although the RMSD score is much better after refinement, the backbones do not look very different. **c** Comparisons of RCI (gray) with flexibility calculated using FIRST for representative models from CNS (blue) and CNW (orange) refinements. The colored bars at the top of each plot show the regular secondary structures: α-helix (red) and β-sheet (blue). The three proteins are (top) the N-terminal domain of VAM3P from *S. cerevisiae* (CNS/CNW model 4, PDB ID 1hs7), a largely helical protein; (middle) a single-domain antibody from *Brucella* (CNS/CNW model 20, PDB ID 1ieh), a largely β-sheet protein, and (bottom) the restriction of telomere capping protein 3 from *S. cerevisiae* (CNS/CNW model 14, PDB ID 1nyn), a mixed α/β protein.

We have therefore carried out a comparison of those CNS and CNW datasets for which there is sufficient (>75%) chemical shift completeness, which comprises a set of 173 ensembles each made up of 25 models (see Supplementary Table 1 for details). From here on we refer to these datasets as CNS75 and CNW75, respectively. In Fig. 2a, the differences in average correlation and RMSD score for each of the 173 ensembles are depicted in a histogram. There is no real improvement in correlation score on refinement in water, with an average improvement of only 1.0. This is expected, as the secondary structure, which ultimately determines the location of peaks and troughs and therefore correlation, changes very little during refinement. As an example, Fig. 2b shows the lack of change in fold for one model. In contrast, RMSD scores are greatly improved, with an average increase of 36.2 and with only one ensemble scoring worse after refinement. This is mostly due to the improvement in hydrogen bonding which acts to rigidify the entire protein. This can be seen in the difference in computed rigidity before and after refinement (Fig. 2c).

### Decoy vs experimental structures

A straightforward way to generate a pool of structures of varying accuracy is to calculate decoys. We used the 3DRobot web server^32^, which begins from a crystal or NMR structure, identifies possible structure scaffolds from a library, assembles them together, and then refines them. The sets of structures generated using 3DRobot are designed to have a high density of structures close to the native state with good hydrogen bonding and compactness, and of high diversity. In other words, they should look like genuine proteins, with good packing and hydrogen bonds, and they should span a range, from structures that closely resemble the native state, to ones that are very different, although still with good packing and hydrogen bonding. These sets therefore allow us to test whether ANSURR can discriminate between structures that are all geometrically good structures, but differ in their accuracy.

**Fig. 3:**
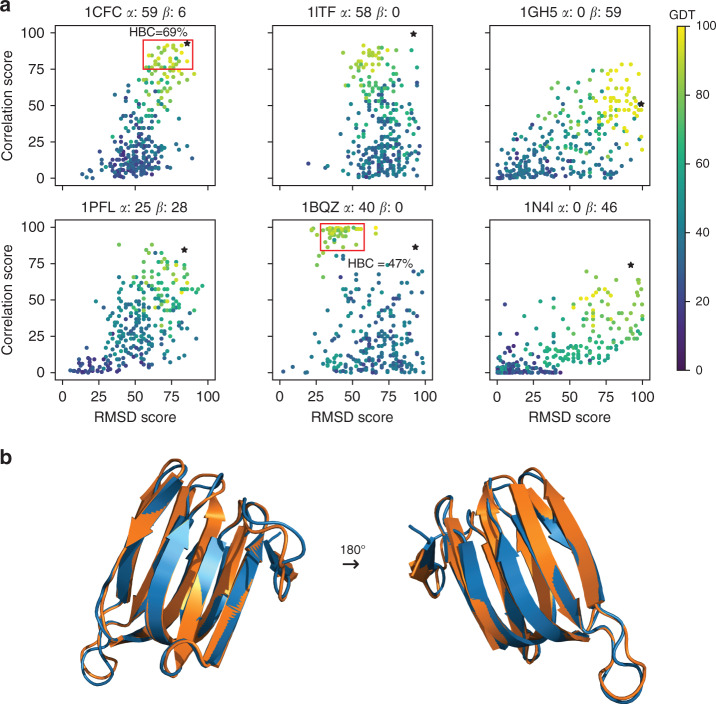
Structural accuracy of decoys. **a** Each plot shows one protein, indicated by its PDB code and the percentage of α-helix and β-sheet in the experimental structure, according to DSSP^61^. The experimental structure is indicated by an asterisk and is the best scoring model in the NMR ensemble, according to our method. The other data show decoys generated by 3DRobot^32^, and color coded by their Global Distance Test (GDT), a measure of similarity to the target^33^, as indicated by the color bar on the right. For two proteins, red boxes indicate the set of decoys used to calculate mean hydrogen bond correctness, as discussed in the text. **b** A comparison of experimental structure (orange) and best decoy (blue) for the protein 1gh5.

For about half (79 of 173) of the ensembles in the CNW75 dataset (see Supplementary Table 2 for a list of the chosen models), we calculated a group of 300 decoys. These decoys were then compared to the experimental structure using a Global Distance Test (GDT), which measures the similarity between two structures, calculated as the largest set of Cα atoms in the model structure falling within a defined cut-off of their position in the test structure, after superimposing the structures^33^. A selection of results is shown in Fig. 3a (results for all 79 sets of decoys are depicted in Supplementary Fig. 1). The score for the experimental structure is indicated by a black asterisk and scores for decoys are circles, colored according to their GDT.

From inspection of the examples shown in Fig. 3a, it can be seen that the experimental model is usually one of the best structures, as one would expect. Also apparent is that as GDT increases (i.e., as decoys become more like the experimental structure), both the validation scores tend towards those of the experimental structure, confirming that our method does specifically validate accuracy. There is a consistent difference between α-helical proteins (e.g., 1itf) and β-sheet proteins (e.g., 1gh5). Helical proteins tend to improve more in their correlation score than in their RMSD score. This seems reasonable: helices are almost always rigid^26^, but not necessarily in the correct location, whereas β-sheet proteins tend to improve more in their RMSD score, because β-sheets can adopt a wide range of local geometries, implying that β-sheet proteins can appear almost correct but have poor hydrogen bonds and thus be much too floppy. Scores for proteins with both α-helical and β-sheet content tend to move in a diagonal, a combination of both effects.

The protein 1bqz presents an interesting example. It is DnaJ, a largely helical protein, and unusually there are many decoys that have a better correlation score but considerably worse RMSD score than the experimental structure, despite most having GDT of around 80 and with some close to 100. However, calculated hydrogen bond correctness scores^34^ i.e., the percentage of hydrogen bonds in the experimental structure that also appear in the decoy, show that these high correlation score decoys (indicated in Fig. 3a with a red box) have poor hydrogen bond geometries (average hydrogen bond correctness of only 47%), and hence a poor RMSD score. By contrast, decoys for 1cfc that approach the accuracy of the experimental structure have good RMSD and correlation scores and have better hydrogen bond geometries (average hydrogen bond correctness of 69%).

Another interesting example is the beta-fold protein 1gh5 (an antifungal protein from *S. tendae*). There are some decoys with better correlation and only marginally worse RMSD scores than the experimental structure, suggesting that they are actually more accurate. Figure 3b compares the experimental structure and best scoring decoy. Immediately obvious (and reassuring) is that at backbone level, both structures are very similar. We note that the experimental structure has a relatively poor correlation score. It is therefore possible that some of the refined decoys genuinely are more accurate: such behavior has been noted before^35^. Inspection of the full dataset in Supplementary Fig. 1 suggests that this is not uncommon. NMR structure refinement is a joint optimization against NMR restraints and known properties of proteins. The observation that some decoys have better scores than NMR structures implies that in some NMR structure calculations, the balance is not yet optimal, and more weight needs to be given to packing and hydrogen bonding for example. We therefore feel that this finding is not a problem with the method: on the contrary, it shows that the method is useful for identifying incompletely refined structures and improving them.

### Comparison between ANSURR and conventional predictors of accuracy

**Fig. 4:**
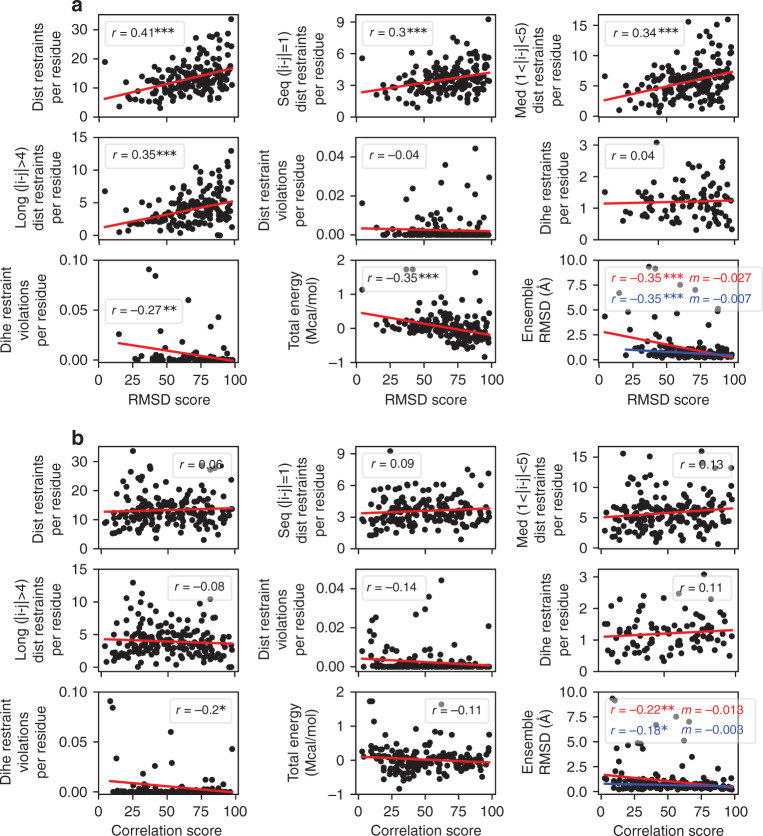
Correlations between conventional NMR-based predictors of accuracy and ANSURR scores. **a** RMSD score, **b** correlation score. For each plot, the line of best fit and the Pearson correlation coefficient are shown. For the comparisons with ensemble RMSD, fits are shown for all points (red) and for only those points with an ensemble RMSD ≤ 2.5 Å (blue). The statistical significance of the correlation coefficient is indicated by ***p < 0.001 **p < 0.01, and *p < 0.05, determined using a two-tailed Pearson test. *p* values are (by row, left to right) **a** 3 × 10^−8^, 7 × 10^−5^, 6 × 10^−6^, 3 × 10^−6^, 0.56, 0.70, 9 × 10^−3^, 2 × 10^−6^, 2 × 10^−6^ (red), 5 × 10^−6^ (blue) and **b** 0.43, 0.22, 0.09, 0.32, 0.07, 0.30, 0.047, 0.13, 4 × 10^−3^ (red), 0.02 (blue).

Conventional predictors of accuracy include the number of restraints per residue used to generate a structure, the number of restraint violations, and the total energy of the structure. The RMSD between models in an ensemble is often used to gauge precision, and by proxy to provide a guide to accuracy. Whilst these measures are expected to be related to accuracy, they do not explicitly determine it. Here we compare these measures to the average RMSD score (Fig. 4a) and correlation score (Fig. 4b) for each ensemble in the CNW75 dataset.

Overall the correlations are much stronger for RMSD score than correlation score. This is not surprising. These predictors largely assess local accuracy, and thus relate to RMSD score better than correlation score.

There is a moderate positive correlation between the number of distance restraints per residue and RMSD score. This is reasonable: a structure with a higher density of distance restraints is expected to be more tightly defined and therefore more (correctly) rigid overall^36^. Categorizing distance restraints according whether they are sequential, medium or long-range reveals a slightly better correlation for medium/long-range restraints than for sequential restraints. This is again expected, as medium/long-range restraints provide more information on protein fold, and for this reason are considered a better predictor of accuracy^37^.

The number of distance restraint violations per residue does not correlate with either validation score. Roughly two thirds of structures do not have any violations at all, because structures are normally refined until there are no, or no significant, violations. It is fairly common practice that restraints that are routinely violated during a structure calculation will be discarded along the way. In fact, programs which automate NMR structure calculation do exactly that. For this reason, restraint violations are clearly not a good predictor of accuracy^8,13,38^.

The number of dihedral restraints per residue does not correlate with either validation score, but dihedral restraint violations do. This is probably because the restraints themselves are relatively weak, so that they do not particularly guide the structure to become more accurate. However, weak negative correlation to dihedral restraint violations suggests that these kinds of restraints successfully flag major issues.

There is a moderate negative correlation to the total energy of the structure. Typically, the selection of the final set of structures to represent the ensemble is based on total energy, and the correlation seen here suggests that this is a reasonable way of identifying good structures.

Both RMSD score and correlation score are negatively correlated with ensemble RMSD suggesting that more precise ensembles do also tend to be more accurate. However, if those ensembles with RMSD larger than 2.5 Å are excluded (blue fit lines) then the gradient becomes almost zero, suggesting that for better structures, ensemble RMSD is a poor guide to accuracy. Similar comments have been made previously^14–17,39^.

In summary, our measures of accuracy match reasonably well to expectations: the number of distance restraints per residue is a fairly good predictor of accuracy, while dihedral restraints, and distance and angle violations, are not. Precision (ensemble RMSD) is a poor predictor of accuracy, while overall energy is surprisingly good as a predictor of accuracy.

### Comparison between ANSURR and geometry-based validation measures

**Fig. 5:**
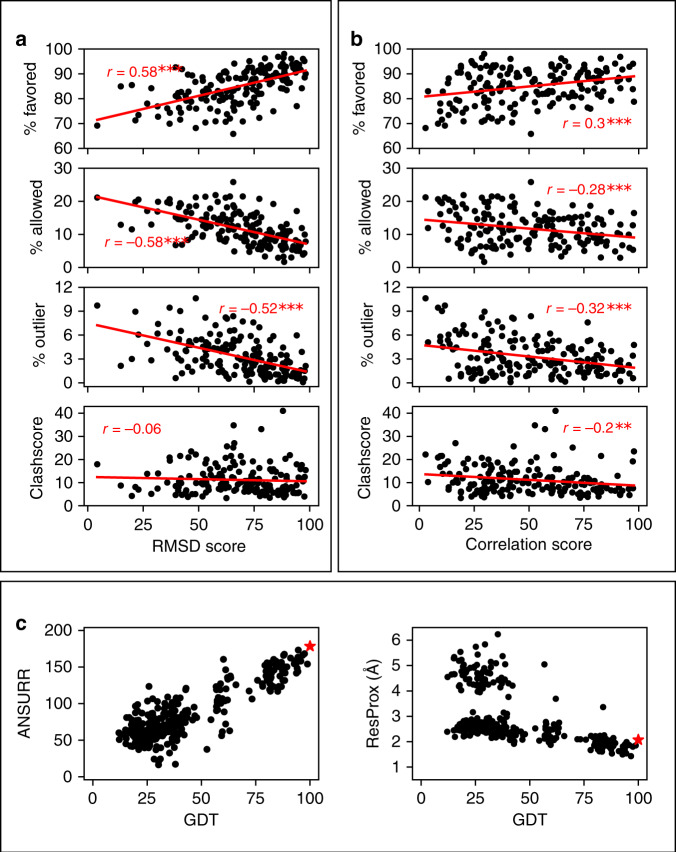
Performance of ANSURR against geometry-based measures. The top part shows correlations between geometry-based measures and **a** RMSD score, **b** correlation score. The statistical significance of the correlation coefficient is indicated by ***p < 0.001, **p < 0.01, and *p < 0.05, determined using a two-tailed Pearson test. *p* values are (top to bottom) **a** 1 × 10^−16^, 4 × 10^−17^, 2 × 10^−13^, 0.44 **b** 8 × 10^−5^, 2 × 10^−4^, 2 × 10^−5^, 8 × 10^−3^. **c** Comparison of ANSURR to ResProx, using 300 decoys generated by 3DRobot for the test PDB file 1cfc. The horizontal axis is the Global Distance Test, a measure of similarity to the test structure (see Fig. 3), which is indicated by the red asterisk. The left box assesses the decoys using ANSURR, where for simplicity we have combined the RMSD score and correlation score into a single sum. There are no decoys with better ANSURR score than the test structure. The right box assesses the same set of decoys using ResProx. There are 57 decoys with better (i.e., lower) ResProx values than the test structure. See Supplementary Fig. 2 for more comparisons.

It is unclear whether a correlation should be expected between geometrical quality and accuracy. However, given that NMR structure calculation is to a large extent an optimization of models, using both NMR-derived restraints and knowledge-derived geometrical factors simultaneously, it is reasonable to expect that an accurate structure should also have good geometrical quality. We therefore compared our validation scores with two widely used indicators of geometrical quality: Ramachandran outliers and clashscore^40^. The program ramalyze (part of the Molprobity suite of validation tools) was used to compute the φ/ψ angles for each residue in the CNW75 dataset and categorize them as either favorable, allowed or outlier. The program clashscore (also part of Molprobity) was used to compute the average number of clashes per 1000 atoms for each ensemble in the CNW75 dataset. In Fig. 5a, b, the results for each ensemble are plotted against RMSD score and correlation score, respectively.

The correlation between Ramachandran distribution and RMSD score is the best for any of the measures presented here. In other words, an ensemble with good Ramachandran distribution (high percentage in the favored category, low percentage in the additionally allowed category, small percentage in the outlier category) is likely to have good accuracy. It seems reasonable to find that the most accurate structures are in general those with the best backbone geometry, as was proposed many years ago^41^.

Geometrical measures have previously been combined together into a consensus quality indicator called Resolution-by-proxy or ResProx, which combines 25 geometrical measures, and has excellent agreement (*R* = 0.92) with X-ray structure resolution^42^. In Fig. 5c we take one PDB structure (1cfc) and generate 300 decoys (i.e., structures with good protein quality, but spanning a range of similarity to the 1cfc structure as assessed by the Global Distance Test), and show that there is a reasonable match between ResProx score and GDT. In other words, structures that are closer to the NMR structure are in general of better geometrical quality. However, we also show that the match is much better for ANSURR: in other words, ANSURR performs much better than a consensus goodness measure based simply on geometrical features. Supplementary Fig. 2 includes results for a range of other proteins, with similar results in all cases.

We have also carried out a similar comparison, but against the consensus measure PROSESS, which combines a wide range of both geometry-based and restraint-based measures, and is thus the closest available consensus test for ANSURR^43^. The PROSESS scores are critically dependent on NOE restraint violations, and are thus subject to the same problems as discussed in the previous section. A more detailed discussion can be found in Supplementary Information.

### Comparison of NMR and X-ray crystal structures

An obvious first test for this method is to compare NMR and X-ray crystal structures. It is important to stress here that because we compare the structures to time-averaged chemical shifts obtained using solution NMR, we are explicitly testing how well the structures compare to the average state of the protein in solution. Crystal structures are almost always based on many more experimental values, and more precisely measured values, than NMR structures. One would therefore inherently expect them to be more accurate, except that crystal structures represent the structure of the protein in a crystalline environment, whereas the NMR chemical shifts measure structural rigidity in solution. We are therefore here making a somewhat unfair, but important, comparison, namely how well X-ray structures represent the structure of a protein in solution.

**Fig. 6:**
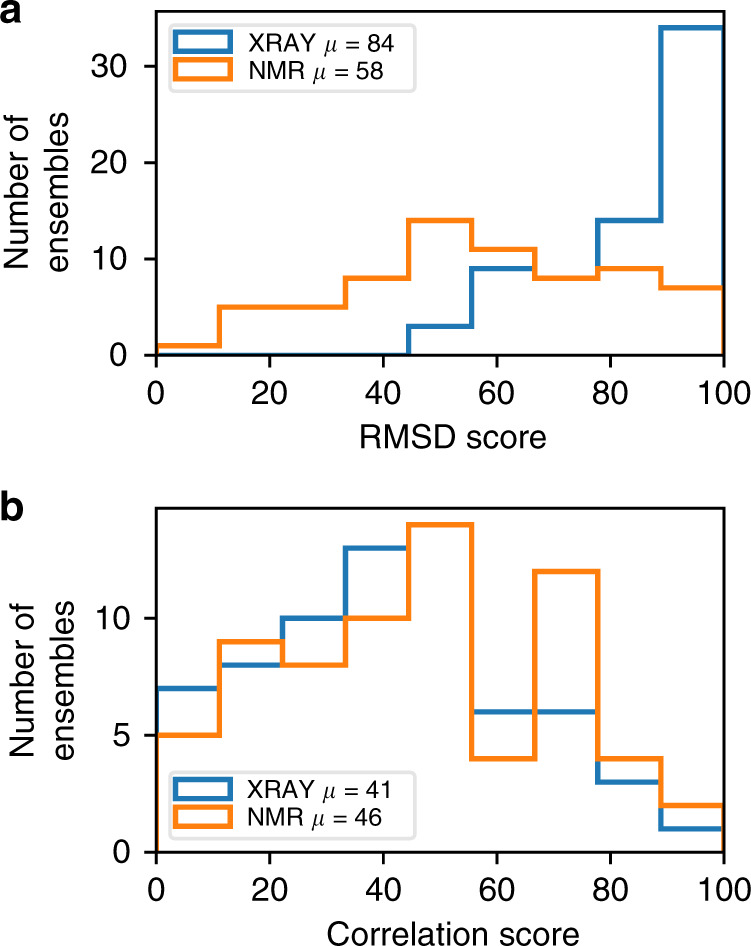
Frequency distributions for X-ray structures (blue) and NMR ensembles (orange) as a function of ANSURR scores. **a** RMSD score and **b** correlation score. The mean values for each score are shown in the inset box.

Here we compare X-ray structures for 68 proteins taken from the set used to train the SHIFTX2 program for predicting chemical shifts^44^ with corresponding NMR structures taken from the PDB (see “Methods” section for details). We validated each structure using our method and averaged the validation scores over each chain for X-ray structures, and each model for NMR ensembles. The results are shown in Fig. 6. The correlation scores for X-ray and NMR structures are very similar. In other words, the locations of rigid and flexible regions, generally representing regular secondary structure in solution, are calculated similarly well by both methods. The slightly lower correlation score for X-ray structures originates from some loops seeming to be too rigid. That is, X-ray structures are missing some peaks in flexibility that should be there according to RCI. Crystal structures are obtained from crystalline arrays, and are usually obtained at cryo-temperatures, both of which will tend to reduce the observed flexibility. There is a large body of evidence^45–47^ that crystal structures obtained at room temperature show much more local variability than do structures obtained at cryo-temperatures, and calculations on lysozyme confirm that the room temperature structures have flexibility that matches the RCI data much better than cryo-temperature structures (Supplementary Note 1 and Supplementary Figs. 3–6). By contrast, in the RMSD score comparison, on average crystal structures are significantly better. When one inspects the data for individual proteins, it is clear that NMR structures are in general much too flexible, particularly in loop regions. This is not unexpected, as NMR structures often have few restraints in loops.

## Discussion

We present a method for determining the accuracy of NMR structures. A range of methods have been proposed previously^10,13,41,48^, including various attempts at an NMR *R* factor^18,19,49–51^. Our method has the merits of being simple, rapid, and in agreement with intuitive expectations. Considering that the first NMR structure of a globular protein was published in 1985^52^, it is remarkable that it has taken this long to come up with a workable measure. The lack of a good measure of accuracy has inhibited researchers from using NMR structures; it is hoped that this method will give users more confidence in the use of structural data from NMR. ANSURR is not a reliable measure of accuracy on its own: as is done for X-ray crystallography, it needs to be combined with other measures, typically geometrical tests.

Because there are no general methods for measuring accuracy, and thus no agreed sets of “good” or “bad” NMR structures, we have been forced to create our own comparisons. Similarly, there are a range of measures that have been proposed for measuring accuracy. In particular, the PDB NMR validation task force^5^ has recommended a set of measures, combining geometrical comparisons and comparisons to input data. These measures are investigated here. We find that the best current indicator of accuracy is a Ramachandran analysis, using either the proportion of residues in the favored region or the proportion of outliers. We find that the RMSD between models in an ensemble is a poor measure of accuracy (though an excellent measure of precision, reinforcing the concept that accuracy and precision are largely independent). Other common restraint-based measures of accuracy, such as restraints per residue^8^ or restraint violations, are also poor measures of accuracy^53^. We suspect that part of the problem is that the route from NOE spectrum to distance restraint contains a large number of user-defined decisions (many of which are increasingly being made by the programs, and are thus becoming even more opaque), so that the link between spectrum and restraint is ill defined.

An interesting conclusion to come from this comparison is that the most common measure of structural similarity, backbone RMSD, misses many of the interesting differences. Structures can look very similar when superimposed on the backbone, but contain large variability in sidechain position and hydrogen bond geometry, which has major impact on docking algorithms and on functional aspects such as allostery, enzyme catalysis^54^, and dynamics.

Now that we have a reliable measure of accuracy, it can be applied to some key problems, for example: (1) how good are the NMR ensembles in the PDB? (2) Can we determine which structures in an ensemble are good, and which are not, and can we therefore improve the ensemble? (3) Is it possible to use experimental NMR data to validate or refine protein structure prediction methods? (4) Can one use these methods to identify local errors in NMR structures? We plan to address these questions in the future.

## Methods

### Random coil index (RCI)

RCI quantifies local (i.e., per residue) protein flexibility by calculating an inverse weighted average of backbone secondary chemical shifts. We calculate RCI essentially as done by Berjanskii and Wishart^22^, though with a few differences. In the originally published method, the weighting coefficients were not normalized. That is, the sum of the weights for different combinations of shifts did not add up to the same value and therefore the baseline rigidity measure could vary when comparing RCI values calculated with different combinations of shifts. We addressed this by simply dividing the sum of weighted secondary shifts by the sum of the weighting coefficients. We therefore compute RCI as:

1 $${\mathrm{RCI}} = \left( {\frac{{A\left| {{\Delta}\delta _{{\mathrm{C}}\alpha }} \right| + B\left| {{\Delta}\delta _{{\mathrm{CO}}}} \right| + C\left| {{\Delta}\delta _{{\mathrm{C}}\beta }} \right| + D\left| {{\Delta}\delta _{\mathrm{N}}} \right| + E\left| {{\Delta}\delta _{{\mathrm{NH}}}} \right| + F\left| {{\Delta}\delta _{{\mathrm{H}}\alpha }} \right|}}{{A + B + C + D + E + F}}} \right)^{\!\!- 1},$$

where the Δ*δ*_I_ are secondary chemical shifts and *A*–*F* are weighting coefficients. Some nuclei (Cα, Cβ) are more descriptive than others (HN, NH) and so have larger weighting coefficients. Missing chemical shifts have a weighting coefficient of zero. Another difference is that we use random coil values and nearest neighbor sequence corrections using data obtained from intrinsically disordered proteins^55^, rather than data based on unfolded peptides or proteins (see e.g., ^56^). A result of these differences is that our approach outputs a value between 0 and 0.2, rather than between 0 and 0.6 as in the originally published method.

We use the set of optimized weighting coefficients for each of the 63 different combinations of backbone chemical shifts as found in the downloadable Python version of RCI http://www.randomcoilindex.com/. For some combinations, we found the similarity between flexibility predicted by RCI and FIRST is significantly decreased suggesting that, in these instances, RCI is a poor predictor of flexibility. Ultimately, the most reliable validation scores are obtained when a full complement of backbone chemical shifts are provided. Our method will allow validation with any combination/completeness of shifts, but the resulting validation score is flagged as less reliable if total chemical shift completeness drops below 75%. For proteins with sufficient chemical shift completeness (≥75%), we assume that residues with completely missing backbone chemical shift assignments are missing because the residues are highly mobile. We assign such residues a secondary chemical shift of zero (i.e., they are assumed to be entirely random coil-like) prior to 3-residue smoothing. However, these data points are not used when calculating validation scores. We note that artificially reducing chemical shift completeness by randomly removing some assignments resulted in worse RMSD and correlation scores, indicating that RCI is more accurate with a greater shift completeness (Supplementary Fig. 7).

### Floppy inclusions and rigid substructure topography (FIRST)

Given a protein structure, FIRST^25^ generates a graph (constraint network) composed of vertices (nodes), which represent atoms; and edges, which represent constraints imposed by the local geometry. Single covalent bonds are modeled by five edges between bonded atoms; double bonds by six; hydrophobic interactions, which are less geometrically constraining, by two; and hydrogen bonds by between one and five, depending on how one chooses to model them. Overall this multigraph represents a generic realization of a molecular body-bar framework in rigidity theory^26^. Typically, rigidity analysis is performed at a range of hydrogen bond energy cut-off values, where hydrogen bonds that meet the cut-off threshold are assigned five edges while weaker interactions are ignored.

Atoms are considered to be rigid bodies each with six degrees of freedom (three position and three orientation). These degrees of freedom are removed as constraints are added between them. One edge removes up to one degree of freedom e.g., a single covalent bond can remove up to five degrees of freedom between the two bonded atoms. FIRST then uses the combinatorial pebble game algorithm (which checks the counting condition prescribed by rigidity theory^57^) to rapidly decompose the graph into maximum rigid clusters and flexible regions, a process known as rigid cluster decomposition. We consider a residue to be rigid if the Cα atom belongs to a rigid cluster that contains at least 15 atoms: this is a useful caveat because it prevents prolines and aromatic residues automatically showing up as rigid.

Relative flexibility is quantified using a process termed hydrogen bond dilution, which is analogous to the thermal denaturation of a protein. Dilution involves incrementally removing edges associated with hydrogen bonds in the graph (weakest to strongest), repeating rigid cluster decomposition and noting the hydrogen bond energy at which the Cα atom of each residue is no longer part of a rigid cluster i.e., becomes flexible. An important benefit of the dilution plot is that the exact energy of each hydrogen bond is not critical to the analysis. We have adapted this slightly, choosing to convert the energies to a Boltzmann population ratio at 298.15 K to represent the probability that a residue is flexible.

### Comparing RCI and FIRST

A simple comparison of RCI and FIRST is not ideal, because the frequency distributions of RCI and FIRST output values are different (Supplementary Fig. 8a, b). The main difference is that RCI is calculated as the inverse of averaged secondary chemical shifts and therefore it is not possible to achieve a RCI value of zero. We decided to rescale RCI values so that the mode RCI value (0.024) becomes “zero” and round up any subsequent negative values. At the other end of the scale, particularly noticeable is a large spike in RCI values at 0.2 which is comprised of terminal residues. A similar spike, also comprised of terminal residues, is present in the frequency distribution of FIRST at Boltzmann population ratio equal to one (i.e., completely flexible at 298.15 K). We therefore decided to scale RCI values so that these spikes align. Subsequent values above one (i.e., apparently more flexible than terminal residues) are rounded down, although such instances are very rare. The equation below outlines how we compute rescaled RCI $\left( {R_{{\mathrm{RCI}}}^\prime } \right)$ from the original RCI values (*R*_RCI_):

2 $$R_{{\mathrm{RCI}}}^\prime = {\mathrm{min}}\left( {\frac{{{\mathrm{max}}(R_{{\mathrm{RCI}}} - 0.024,0)}}{{0.2 - 0.024}},1} \right).$$

Comparing the frequency distribution of the rescaled RCI and FIRST output values shows good agreement (Supplementary Fig. 8c, d).

### Validation scores

RCI and FIRST are compared using two different measures. One is the correlation, calculated using a Spearman rank correlation coefficient. The other is the root mean square deviation (RMSD), calculated as:

3 $${\mathrm{RMSD}} = \sqrt {\frac{{{\sum} {\left( {R_{{\mathrm{RCI}}}^\prime - R_{{\mathrm{FIRST}}}} \right)^2} }}{N}},$$

where *N* is the number of residues in the protein, $R_{{\mathrm{RCI}}}^\prime$ is the local rigidity computed with RCI and rescaled as described above, and *R*_FIRST_ is the local rigidity computed with FIRST. The numerical values of correlation score and RMSD score are reported as the percentiles relative to a reference dataset formed of structures from the CNS and CNW datasets from the RECOORD recalculated structure database, which provide a representative selection of different fold types, before and after explicit solvent refinement.

### Dataset of comparable X-ray and NMR structures

To build a dataset of comparable X-ray and NMR structures, we made use of the set of X-ray structures that were used to train the SHIFTX2 program for predicting chemical shifts^44^. This set comprises 197 high-resolution and high-quality structures, which are representative of different fold types. We extracted structures which had corresponding NMR structures in the PDB, and backbone chemical shift completeness of at least 75%. Our final dataset consisted of 80 X-ray structures and 121 corresponding NMR structures for 68 different proteins. PDB and BMRB IDs are provided in Supplementary Table 3.

X-ray structures required some processing. If the structure contained multiple conformations (typical in high resolution X-ray structures), then we only considered the first of these as they appeared in the PDB file. Missing atoms and small breaks in the protein structure were identified using an in-house program and fixed using MODELLER^58^. MODELLER was also used to replace non-standard residues related to conditions required for crystallization (e.g., selenomethionine was replaced with methionine). Structures were protonated using REDUCE with the option to optimize adjustable groups^59^.

### Reporting summary

Further information on research design is available in the Nature Research Reporting Summary linked to this article.

## Supplementary information

Supplementary Information

Peer Review File

Reporting Summary

## Data Availability

Source data are listed in Supplementary Information and are from publicly available databases: specifically, the Protein Data Bank (www.rcsb/org), Biological Magnetic Resonance Bank (BMRB: www.bmrb.io) and RECOORD (www.ebi.ac.uk/pdbe/recalculated-nmr-data). The accession codes of PDB and BMRB entries used in this study are listed in the Supplementary Information file. Data supporting the findings of this work are available within the paper and its Supplementary Information. The datasets generated and analysed during the current study are available from the corresponding author (MPW) upon request.

## Code availability

The program and associated documentation can be downloaded from github.com/nickjf/ANSURR, 10.5281/zenodo.4161586^60^. A typical calculation on an ensemble of 20 models for a 150-residue protein takes less than a minute.

## References

[1] Berman HM (2000). The protein data bank. Nucleic Acids Res..

[2] Read RJ (2011). A new generation of crystallographic validation tools for the Protein Data Bank. Structure.

[3] Henderson R (2012). Outcome of the first electron microscopy validation task force meeting. Structure.

[4] Trewhella J (2013). Report of the wwPDB small-angle scattering task force: data requirements for biomolecular modeling and the PDB. Structure.

[5] Montelione GT (2013). Recommendations of the wwPDB NMR validation task force. Structure.

[6] Gore S (2017). Validation of structures in the Protein Data Bank. Structure.

[7] Brunger AT (1992). Free R-value: a novel statistical quantity for assessing the accuracy of crystal structures. Nature.

[8] Snyder DA, Bhattacharya A, Huang YPJ, Montelione GT (2005). Assessing precision and accuracy of protein structures derived from NMR data. Proteins.

[9] Vuister GW, Fogh RH, Hendrickx PMS, Doreleijers JF, Gutmanas A (2014). An overview of tools for the validation of protein NMR structures. J. Biomol. NMR.

[10] Spronk CAEM, Nabuurs SB, Krieger E, Vriend G, Vuister GW (2004). Validation of protein structures derived by NMR spectroscopy. Progr. NMR Spectrosc..

[11] Nabuurs SB, Spronk CAEM, Vuister GW, Vriend G (2006). Traditional biomolecular structure determination by NMR spectroscopy allows for major errors. PLos Comput. Biol..

[12] Brünger AT, Clore GM, Gronenborn AM, Saffrich R, Nilges M (1993). Assessing the quality of solution nuclear magnetic resonance structures by complete cross-validation. Science.

[13] Huang YJ, Rosato A, Singh G, Montelione GT (2012). RPF: a quality assessment tool for protein NMR structures. Nucleic Acids Res..

[14] Williamson MP, Kikuchi J, Asakura T (1995). Application of ^1^H NMR chemical shifts to measure the quality of protein structures. J. Mol. Biol..

[15] Zhao DQ, Jardetzky O (1994). An assessment of the precision and accuracy of protein structures determined by NMR: dependence on distance errors. J. Mol. Biol..

[16] Saccenti E, Rosato A (2008). The war of tools: how can NMR spectroscopists detect errors in their structures?. J. Biomol. NMR.

[17] Spronk CAEM (2003). The precision of NMR structure ensembles revisited. J. Biomol. NMR.

[18] Gronwald W (2000). RFAC, a program for automated NMR R-factor estimation. J. Biomol. NMR.

[19] Gronwald W (2007). AUREMOL-RFAC-3D, combination of R-factors and their use for automated quality assessment of protein solution structures. J. Biomol. NMR.

[20] Wüthrich K (1986). NMR of Proteins and Nucleic Acids.

[21] Wishart DS (2011). Interpreting protein chemical shift data. Prog. Nucl. Magn. Reson. Spectrosc..

[22] Berjanskii MV, Wishart DS (2008). Application of the random coil index to studying protein flexibility. J. Biomol. NMR.

[23] Berjanskii MV, Wishart DS (2005). A simple method to predict protein flexibility using secondary chemical shifts. J. Am. Chem. Soc..

[24] Sljoka A, Wilson D (2013). Probing protein ensemble rigidity and hydrogen-deuterium exchange. Phys. Biol..

[25] Jacobs DJ, Rader AJ, Kuhn LA, Thorpe MF (2001). Protein flexibility predictions using graph theory. Proteins.

[26] Whiteley W (2005). Counting out to the flexibility of molecules. Phys. Biol..

[27] Nederveen AJ (2005). RECOORD: a recalculated coordinate database of 500+ proteins from the PDB using restraints from the BioMagResBank. Proteins.

[28] Brunger AT (1998). Crystallography & NMR system: a new software suite for macromolecular structure determination. Acta Cryst. D.

[29] Güntert P (2003). Automated NMR protein structure calculation. Progr. NMR Spectrosc..

[30] Linge JP, Habeck M, Rieping W, Nilges M (2003). ARIA: automated NOE assignment and NMR structure calculation. Bioinformatics.

[31] Linge JP, Williams MA, Spronk CAEM, Bonvin AMJJ, Nilges M (2003). Refinement of protein structures in explicit solvent. Proteins.

[32] Deng H, Jia Y, Zhang Y (2016). 3DRobot: automated generation of diverse and well-packed protein structure decoys. Bioinformatics.

[33] Zemla A (2003). LGA: a method for finding 3D similarities in protein structures. Nucleic Acids Res..

[34] Keedy DA (2009). The other 90% of the protein: Assessment beyond the Cαs for CASP8 template-based and high-accuracy models. Proteins.

[35] Mao B, Tejero R, Baker D, Montelione GT (2014). Protein NMR structures refined with Rosetta have higher accuracy relative to corresponding X-ray crystal structures. J. Am. Chem. Soc..

[36] Clore GM, Robien MA, Gronenborn AM (1993). Exploring the limits of precision and accuracy of protein structures determined by nuclear magnetic resonance spectroscopy. J. Mol. Biol..

[37] Nabuurs SB (2003). Quantitative evaluation of experimental NMR restraints. J. Am. Chem. Soc..

[38] Huang YPJ (2005). An integrated platform for automated analysis of protein NMR structures. Methods Enzymol..

[39] Simon K, Xu J, Kim C, Skrynnikov N (2005). Estimating the accuracy of protein structures using residual dipolar couplings. J. Biomol. NMR.

[40] Chen VB (2010). MolProbity: all-atom structure validation for macromolecular crystallography. Acta Crystallogr. Sect. D.

[41] Laskowski RA, Rullmann JAC, MacArthur MW, Kaptein R, Thornton JM (1996). AQUA and PROCHECK-NMR: Programs for checking the quality of protein structures solved by NMR. J. Biomol. NMR.

[42] Berjanskii M, Zhou J, Liang Y, Lin G, Wishart DS (2012). Resolution-by-proxy: a simple measure for assessing and comparing the overall quality of NMR protein structures. J. Biomol. NMR.

[43] Berjanskii M (2010). PROSESS: a protein structure evaluation suite and server. Nucleic Acids Res..

[44] Han B, Liu Y, Ginzinger SW, Wishart DS (2011). SHIFTX2: significantly improved protein chemical shift prediction. J. Biomol. NMR.

[45] Tilton RF, Dewan JC, Petsko GA (1992). Effects of temperature on protein structure and dynamics: X-ray crystallographic studies of the protein ribonuclease-A at 9 different temperatures from 98 K to 320 K. Biochemistry.

[46] Fraser JS (2011). Accessing protein conformational ensembles using room-temperature X-ray crystallography. Proc. Natl Acad. Sci. USA.

[47] Halle B (2004). Biomolecular cryocrystallography: Structural changes during flash-cooling. Proc. Natl Acad. Sci. USA.

[48] Doreleijers JF, Rullmann JAC, Kaptein R (1998). Quality assessment of NMR structures: a statistical survey. J. Mol. Biol..

[49] Gonzalez C, Rullmann JAC, Bonvin AMJJ, Boelens R, Kaptein R (1991). Toward an NMR *R* factor. J. Magn. Reson..

[50] Thomas PD, Basus VJ, James TL (1991). Protein structure determination using distances from 2-dimensional nuclear Overhauser effect experiments: effect of approximations on the accuracy of derived structures. Proc. Natl Acad. Sci. USA.

[51] Withka JM, Srinivasan J, Bolton PH (1992). Problems with, and alternatives to, the NMR *R* factor. J. Magn. Reson..

[52] Williamson MP, Havel TF, Wüthrich K (1985). Solution conformation of proteinase inhibitor IIA from bull seminal plasma by ^1^H nuclear magnetic resonance and distance geometry. J. Mol. Biol..

[53] Vranken WF (2014). NMR structure validation in relation to dynamics and structure determination. Prog. Nucl. Magn. Reson. Spectrosc..

[54] Kim TH (2017). The role of dimer asymmetry and protomer dynamics in enzyme catalysis. Science.

[55] Tamiola K, Acar B, Mulder FAA (2010). Sequence-specific random coil chemical shifts of intrinsically disordered proteins. J. Am. Chem. Soc..

[56] Schwarzinger S (2001). Sequence-dependent correction of random coil NMR chemical shifts. J. Am. Chem. Soc..

[57] Katoh N, Tanigawa S (2011). A proof of the molecular conjecture. Discret. Comput. Geom..

[58] Webb B, Sali A (2016). Comparative protein structure modeling using MODELLER. Curr. Protoc. Protein Sci..

[59] Word JM, Lovell SC, Richardson JS, Richardson DC (1999). Asparagine and glutamine: using hydrogen atom contacts in the choice of side-chain amide orientation. J. Mol. Biol..

[60] Fowler, N. J., Sljoka, A. & Williamson, M. P. A method for validating the accuracy of NMR protein structures. GitHub.com/nickjf/ANSURR 10.5281/zenodo.4161586 (2020). 10.1038/s41467-020-20177-1 PMC7749147 33339822

[61] Kabsch W, Sander C (1983). Dictionary of protein secondary structure: pattern recognition of hydrogen-bonded and geometrical features. Biopolymers.

