# Supplementary material for: A method for validating the accuracy of NMR protein structures
Source: Nat Commun. 2020 Dec 18;11:6321. doi: 10.1038/s41467-020-20177-1 (PMC7749147; doi:10.1038/s41467-020-20177-1)
Supplement: Supplementary file 1 — Supplementary Information [file 41467_2020_20177_MOESM1_ESM.pdf]

A method for validating the accuracy of NMR protein structures

Nicholas J. Fowler[1], Adnan Sljoka[2,3] and Mike P. Williamson[1]
[1]Dept of Molecular Biology and Biotechnology, University of Sheffield, UK. [2]RIKEN Center for Advanced Intelligence Project, RIKEN, 1-4-1 Nihombashi, Chuo-ku, Tokyo, 103-0027 Japan. [3]Dept of Chemistry, University of Toronto, UTM, 3359 Mississauga Road North, Mississauga, ON, L5L 1C6, Canada

Supplementary Table 1. PDB IDs for 173 ensembles from the RECOORD CNS and CNW datasets with chemical shift completeness ≥ 75 %.

1A5J	1F2H	1JQR	1N88
1B22	1F3Y	1JR6	1N91
1B2T	1F43	1JRM	1ND9
1B4R	1F53	1JW2	1NOR
1B64	1FMM	1JW3	1NR3
1B75	1FO7	1JWE	1NWB
1BCN	1FR0	1JYT	1NWV
1BJX	1G03	1JZU	1NXI
1BLR	1G11	1K0S	1NY4
1BNO	1G4F	1K0X	1NY9
1BO0	1G6J	1K1C	1NZP
1BQZ	1G9L	1K5O	1O1W
1C05	1GA3	1K8B	1OCA
1C06	1GH5	1K8O	1OMT
1C3T	1H2O	1K9C	1OMU
1C54	1H3Z	1KFT	1ONB
1CEJ	1H95	1KHM	1OP1
1CEY	1HS7	1KKG	1PBU
1CFC	1HX7	1KMA	1PFL
1CFE	1HY8	1KMD	1PLO
1CMZ	1I42	1KOT	1PN5
1COK	1I5J	1KRW	1PUX
1CX1	1ICH	1KTM	1Q27
1CZ5	1IEH	1L1P	1Q2N
1D1N	1IFW	1L7B	1Q59
1D3Z	1IQO	1L7Y	1QHK
1D8B	1IRZ	1LG4	1QND
1DC7	1ITF	1LM0	1QXF
1DCJ	1IX5	1LS4	1SPY
1DD2	1J0T	1M12	1SSN
1DMO	1J8I	1M2F	1SUH
1DOQ	1J8K	1M5Z	1TBD
1DS9	1JAS	1M7T	1UD7
1DU2	1JDQ	1M94	1XNA
1DV5	1JE4	1MG8	2CPB
1E17	1JE9	1MJD	2CPS
1E41	1JFJ	1MKE	2EZA
1EGX	1JFN	1MP1	2EZH
1EIH	1JGK	1MUT	2MOB
1EWW	1JH3	1MZK	3BDO
1EZA	1JI8	1N3G	3PDZ
1EZO	1JJG	1N4I	
1EZP	1JNS	1N6U	
1EZY	1JOR	1N6Z	

Supplementary Table 2. 79 models from the RECOORD dataset used to generate decoys.

PDB ID	Model number
1A5J	18
1B2T	1
1B4R	18
1B64	12
1B75	16
1BJX	4
1BQZ	11
1CFC	20
1CMZ	4
1COK	8
1CX1	18
1DC7	4
1DD2	12
1DMO	22
1DOQ	3
1E17	22
1EGX	21
1EWW	13
1EZA	11
1F2H	8
1F43	21
1F53	14
1FO7	1
1G03	13
1G11	2
1G4F	13
1GA3	7
1GH5	10
1H95	17
1HX7	15
1ICH	17
1IEH	20
1IFW	14
1IRZ	22
1ITF	13
1IX5	1
1J8I	10
1J8K	5
1JE9	8
1JJG	20

PDB ID	Model number
1JNS	15
1JOR	15
1JR6	9
1JWE	11
1JZU	5
1K0X	24
1K5O	1
1K9C	4
1KKG	7
1KMA	9
1KMD	22
1KRW	22
1L7Y	7
1LG4	14
1LM0	13
1M12	10
1M2F	12
1M7T	10
1MJD	4
1MP1	16
1N3G	2
1N4I	14
1N6U	23
1N6Z	19
1NOR	20
1NXI	5
1PBU	10
1PFL	21
1PN5	2
1Q2N	7
1SPY	3
1SUH	10
1TBD	3
1UD7	4
1XNA	5
2CPB	9
2EZA	3
2EZH	4
3PDZ	19

Supplementary Figure 1. Validation scores computed for each target structure in supplementary table 2 (black asterisk) and 300 decoy structures (circles colored according to GDT). Each plot shows one protein, indicated by PDB ID and the percentage of α-helix and β-sheet in the target structure.

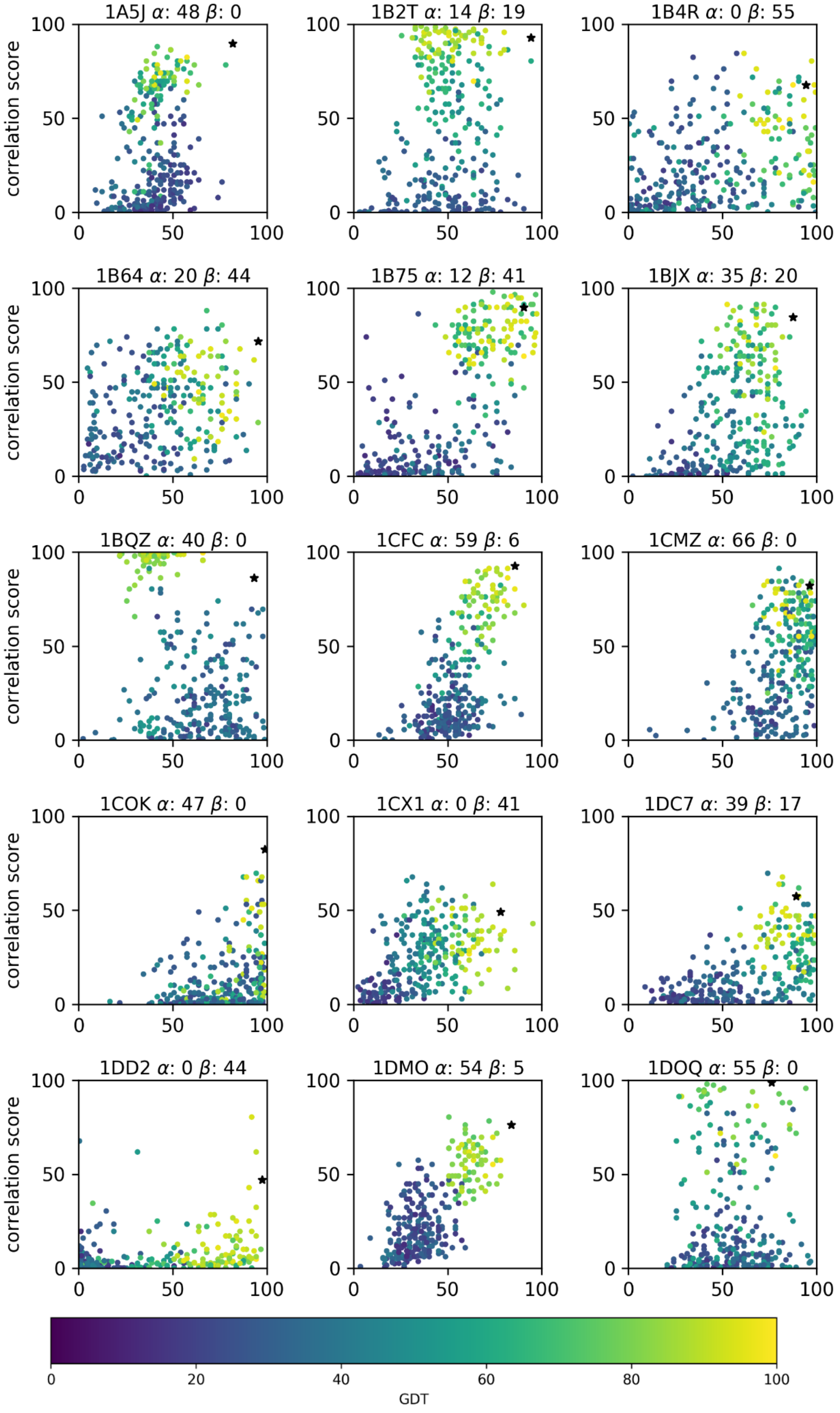

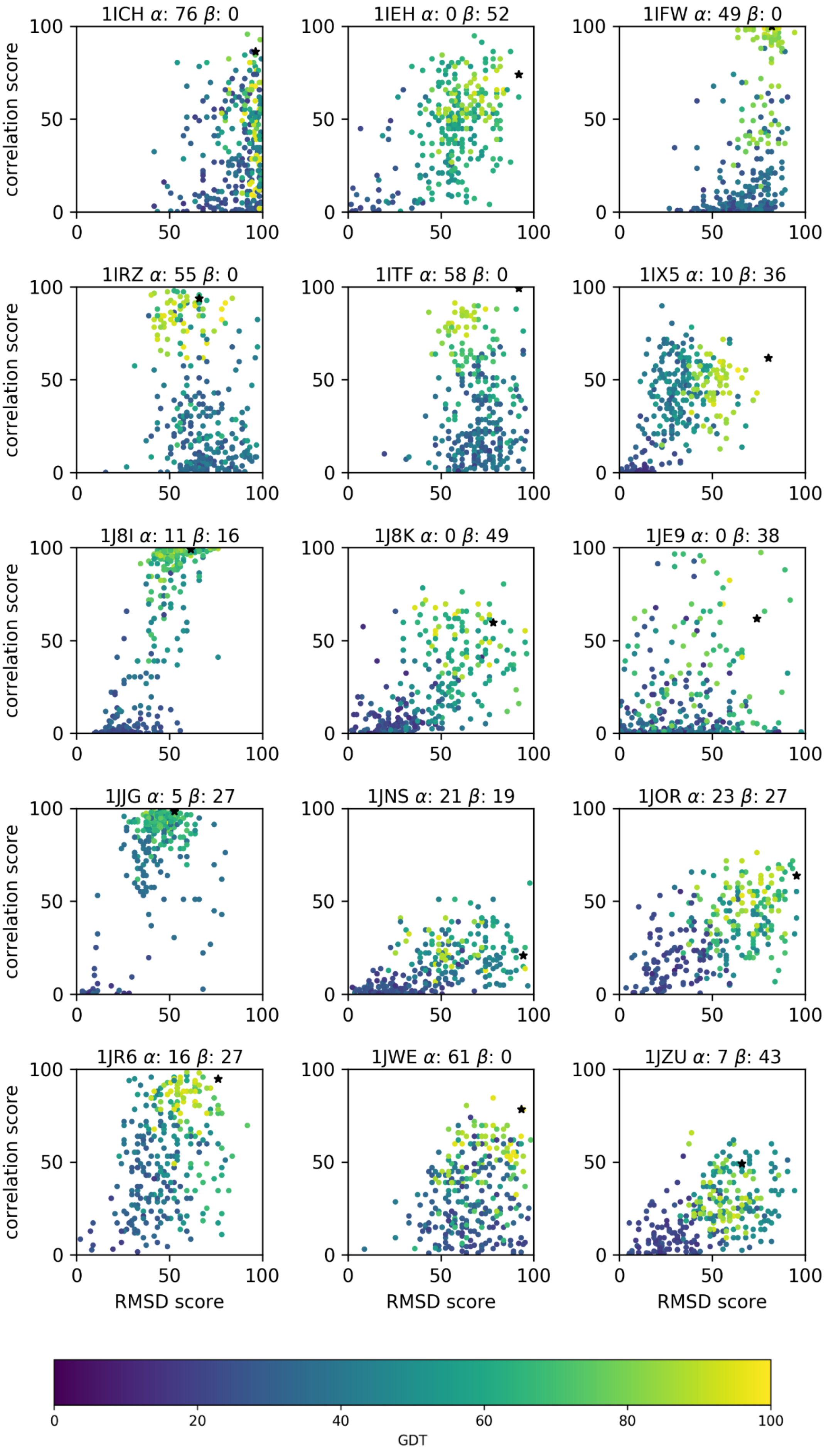
1ICH α: 76 β: 0
1IEH α: 0 β: 52
1IFW α: 49 β: 0
1IRZ α: 55 β: 0
1ITF α: 58 β: 0
1IX5 α: 10 β: 36
1J8I α: 11 β: 16
1J8K α: 0 β: 49
1JE9 α: 0 β: 38
1JJG α: 5 β: 27
1JNS α: 21 β: 19
1JOR α: 23 β: 27
1JR6 α: 16 β: 27
1JWE α: 61 β: 0
1JZU α: 7 β: 43
correlation score
RMSD score
GDT
0
20
40
60
80
100

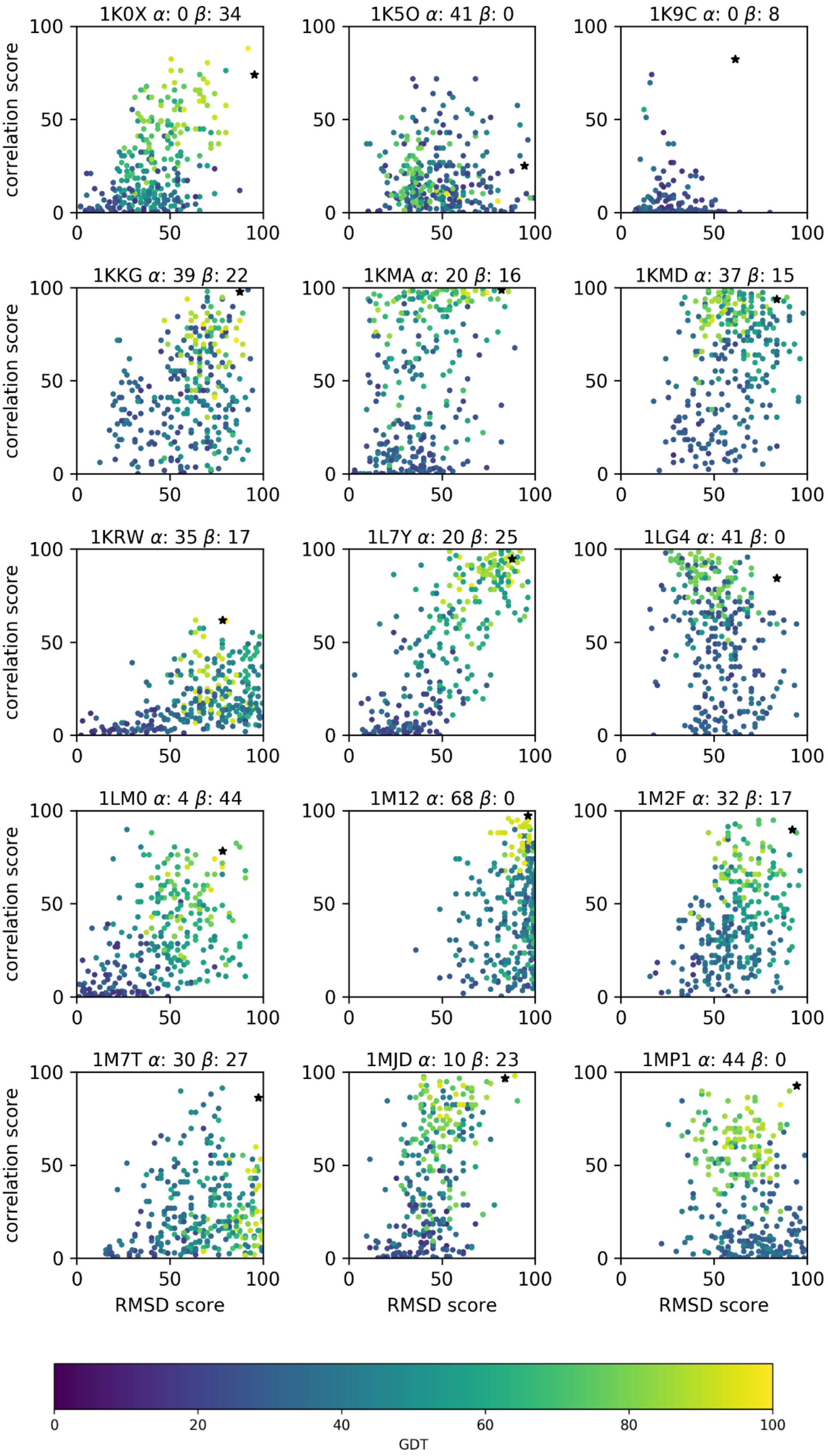
1K0X α: 0 β: 34
1K5O α: 41 β: 0
1K9C α: 0 β: 8
1KKG α: 39 β: 22
1KMA α: 20 β: 16
1KMD α: 37 β: 15
1KRW α: 35 β: 17
1L7Y α: 20 β: 25
1LG4 α: 41 β: 0
1LM0 α: 4 β: 44
1M12 α: 68 β: 0
1M2F α: 32 β: 17
1M7T α: 30 β: 27
1MJD α: 10 β: 23
1MP1 α: 44 β: 0
correlation score
RMSD score
0
50
100
0
20
40
60
80
100
GDT

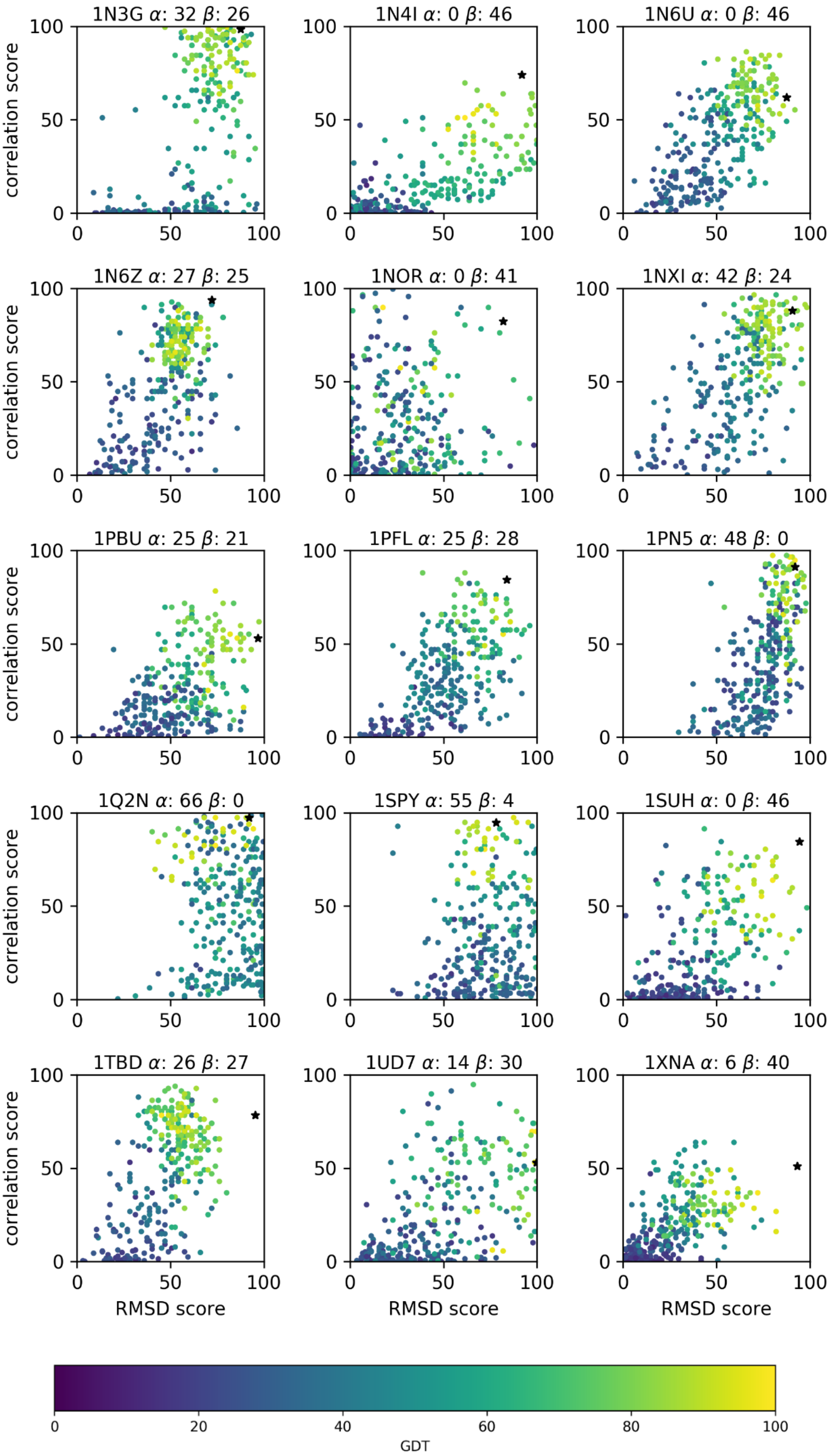

1N3G α: 32 β: 26
1N4I α: 0 β: 46
1N6U α: 0 β: 46
1N6Z α: 27 β: 25
1NOR α: 0 β: 41
1NXI α: 42 β: 24
1PBU α: 25 β: 21
1PFL α: 25 β: 28
1PN5 α: 48 β: 0
1Q2N α: 66 β: 0
1SPY α: 55 β: 4
1SUH α: 0 β: 46
1TBD α: 26 β: 27
1UD7 α: 14 β: 30
1XNA α: 6 β: 40
correlation score
RMSD score
0
50
100
0
20
40
60
80
100
GDT

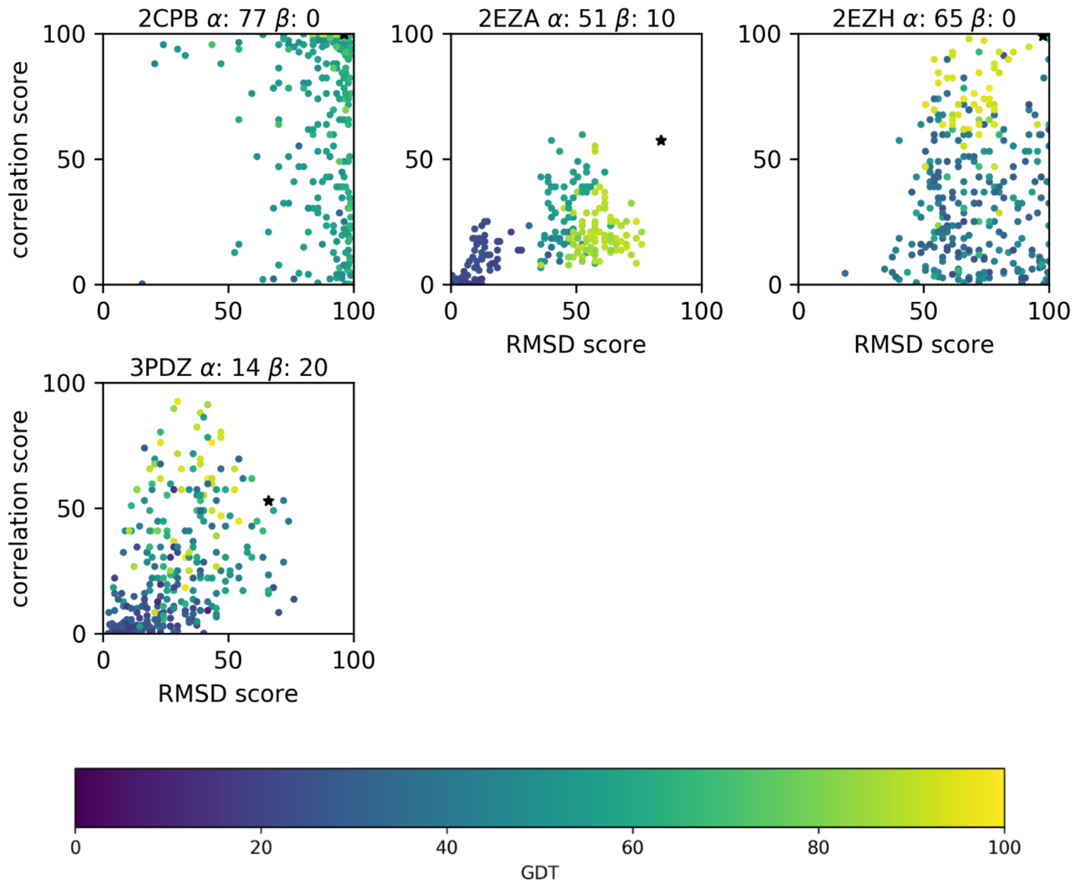

2CPB α: 77 β: 0
2EZA α: 51 β: 10
2EZH α: 65 β: 0
3PDZ α: 14 β: 20
correlation score
RMSD score
0
50
100
0
20
40
60
80
100
GDT

Supplementary Figure 2. Comparison of ANSURR to two consensus measures of accuracy, namely ResProx[1] and PROSESS[2]. ResProx uses a panel of 25 geometrical measures, while PROSESS uses geometrical measures plus back-calculated chemical shifts and NOE distance violations. For the comparisons, we took six target NMR structures from PDB, and for each structure generated 300 decoys using 3DRobot[3], which span a continuous range of similarity to the target, including structures that are closely similar to the target (so similar that a comparison of backbone structures looks virtually identical). For PROSESS, structures had to be submitted individually to the web server, so we randomly selected 30 decoys with a range of GDT values from the set of 300, to speed up the calculations. In each case, the structural accuracy of the panel of decoys is measured using the Global Distance Test (GDT)[4], which measures the percentage accuracy of each decoy compared to the target NMR structure, denoted by the red asterisk (see Fig. 3 of main text for further discussion).

For ANSURR (column 1), the vertical axis is the sum of RMSD score and correlation score. This is not our preferred presentation of ANSURR output, which is to display RMSD and correlation scores separately on a two-dimensional plot; the summed value is simply for convenience. The decoys are shown to scale roughly linearly with GDT, such that decoys with similar structures to the starting structure have similar ANSURR score. Column 2 assesses the same set of decoys using ResProx. The correlation with GDT is less clear; in particular, there is a significant proportion of structures with ResProx scores better than the NMR structure. It is not surprising that ResProx should do less well, because it only considers geometrical quality. Column 3 shows the PROSESS score. PROSESS returns low scores for everything except the target structure, with little discrimination between structures. This is because the PROSESS score is dominated by NOE restraint violations, and is thus very sensitive to exactly what distance restraints are compared. We suspect that if the decoys were refined against the NMR restraints, their PROSESS scores would improve dramatically but the lack of discrimination between good and bad structures would remain. This comparison is therefore not a fair test of whether PROSESS works, because we are using it in a way for which it was not designed. In column 4, we therefore report one component of the distance violation score, namely the root-mean-square NOE violation, which is much less sensitive to individual NOE violations. It is clear that this is a more discriminatory measure than the raw PROSESS score, but for most of the proteins it reaches a plateau, beyond which it is not a clear guide to the accuracy of the decoys. We feel that this is a further indication that NOE violations provide a poor guide to the accuracy of a structure, because they are interpreted data: small changes in user interpretation of the NMR spectra can affect the NOE restraint list in significant ways, without any great effect on the underlying structure accuracy calculated from them.

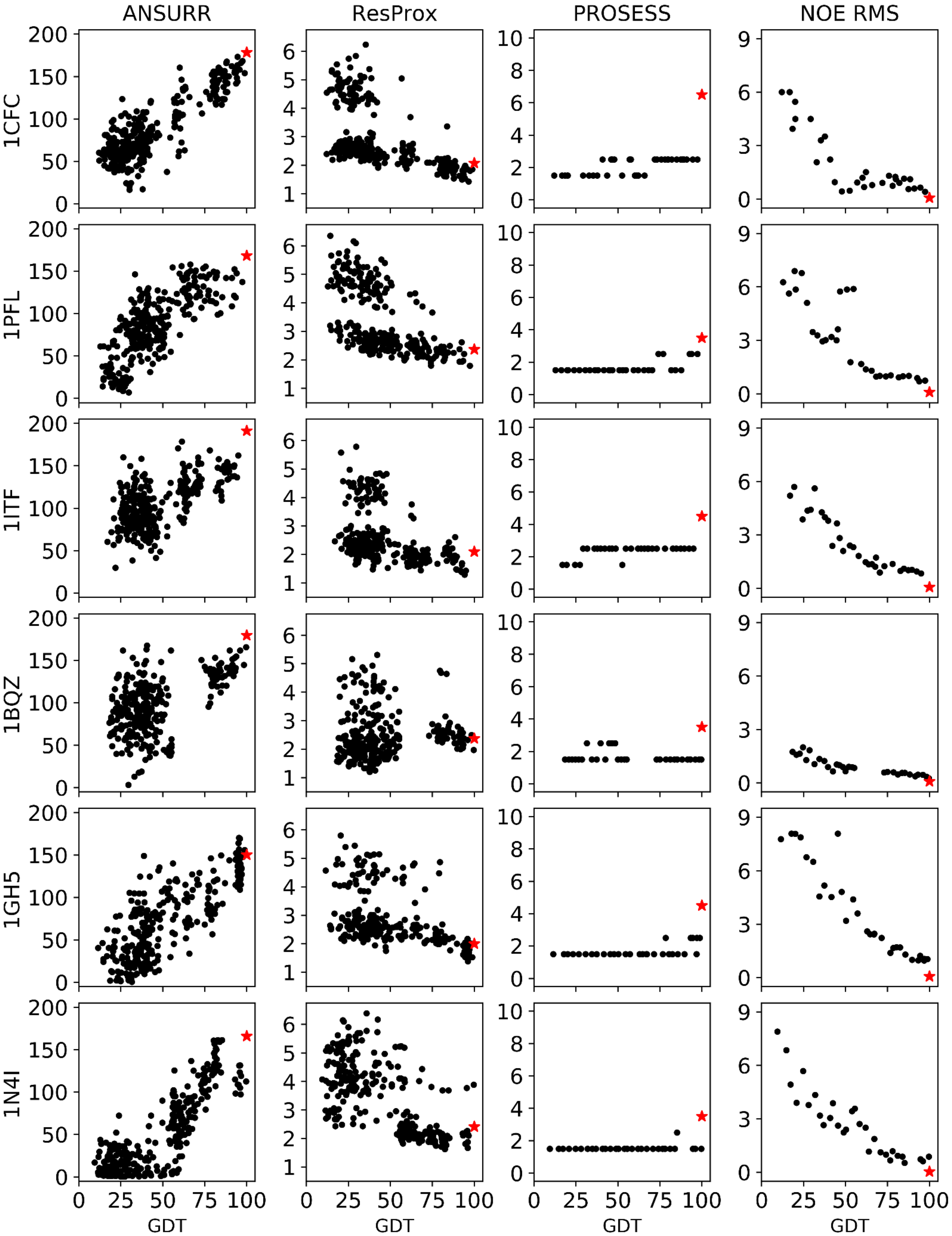
ANSURR
ResProx
PROSESS
NOE RMS
1CFC
1PFL
1ITF
1BQZ
1GH5
1N4I
GDT
GDT
GDT
GDT

Supplementary Note 1

Validation of lysozyme X-ray structures obtained at 100 K and 278 K.

Here we compare X-ray structures of hen egg-white lysozyme obtained at 100 K and 278 K. We chose a set of structures deposited by the same research group for which crystal preparation and data collection were performed by following the same protocol. Structures were downloaded from the PDB (PDB IDs for structures obtained at 100 K: 5KXK, 5KXL, 5KXM, 5KXN; and at 278 K: 5KXO, 5KXP, 5KXR, 5KXS ,5KXT, 5KXW, 5KXX, 5KXY, 5KXZ, 5KY1) and processed in the same way as described in Methods - Dataset of comparable X-ray and NMR structures. Backbone chemical shifts were extracted from two sets deposited to the BMRB (H shifts from BMRB ID 4562 and N/C shifts from BMRB ID 4831). We then validated each structure using ANSURR. The results presented in supplementary Fig. 3 show that the structures obtained at 278 K are better. That is to say, that flexibilities predicted for those structures are a better match to those predicted from chemical shifts obtained in solution at room temperature. These results suggest that X-ray structures obtained at cryogenic temperatures are more rigid than at room temperature. In figures 4 and 5, we compare flexibility predicted by RCI and FIRST for each structure at 100 K and 278 K, respectively. Noticeable is that for structures obtained at 100 K there are many missing peaks in flexibility that should be present according to RCI. Structures obtained at 278 K have many more of these peaks in flexibility. Backbone superposition of the structures shows that the fold is essentially identical at both temperatures (Fig. 6). From inspection of the structures, the over-rigidification of structures obtained at 100 K manifests as additional and/or slightly stronger hydrogen bonds in loop regions.

Supplementary Fig. 3. Validation scores for lysozyme X-ray structures obtained at 100 K (blue) and 278 K (orange).

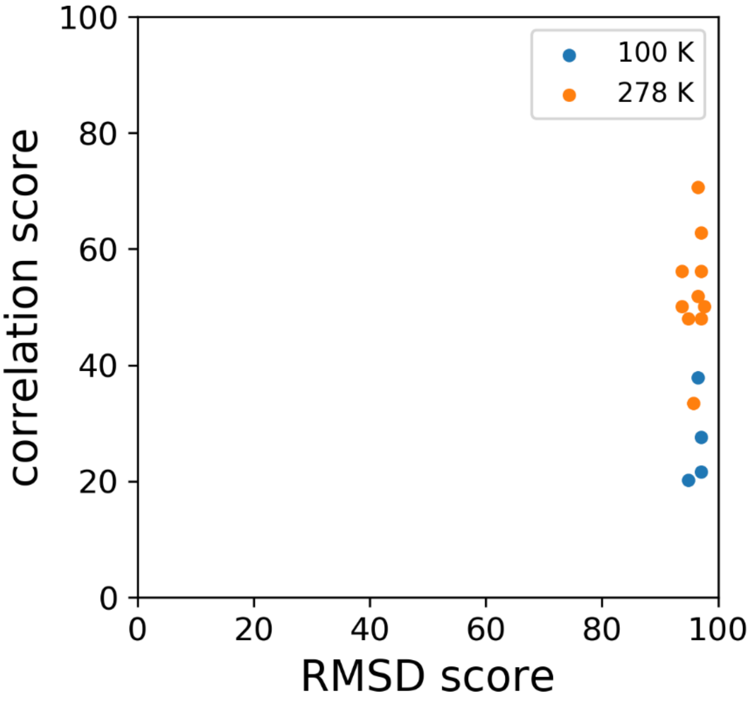

Supplementary Fig. 4. Comparison of flexibility predicted by RCI and FIRST for lysozyme X-ray structures obtained at 100 K.

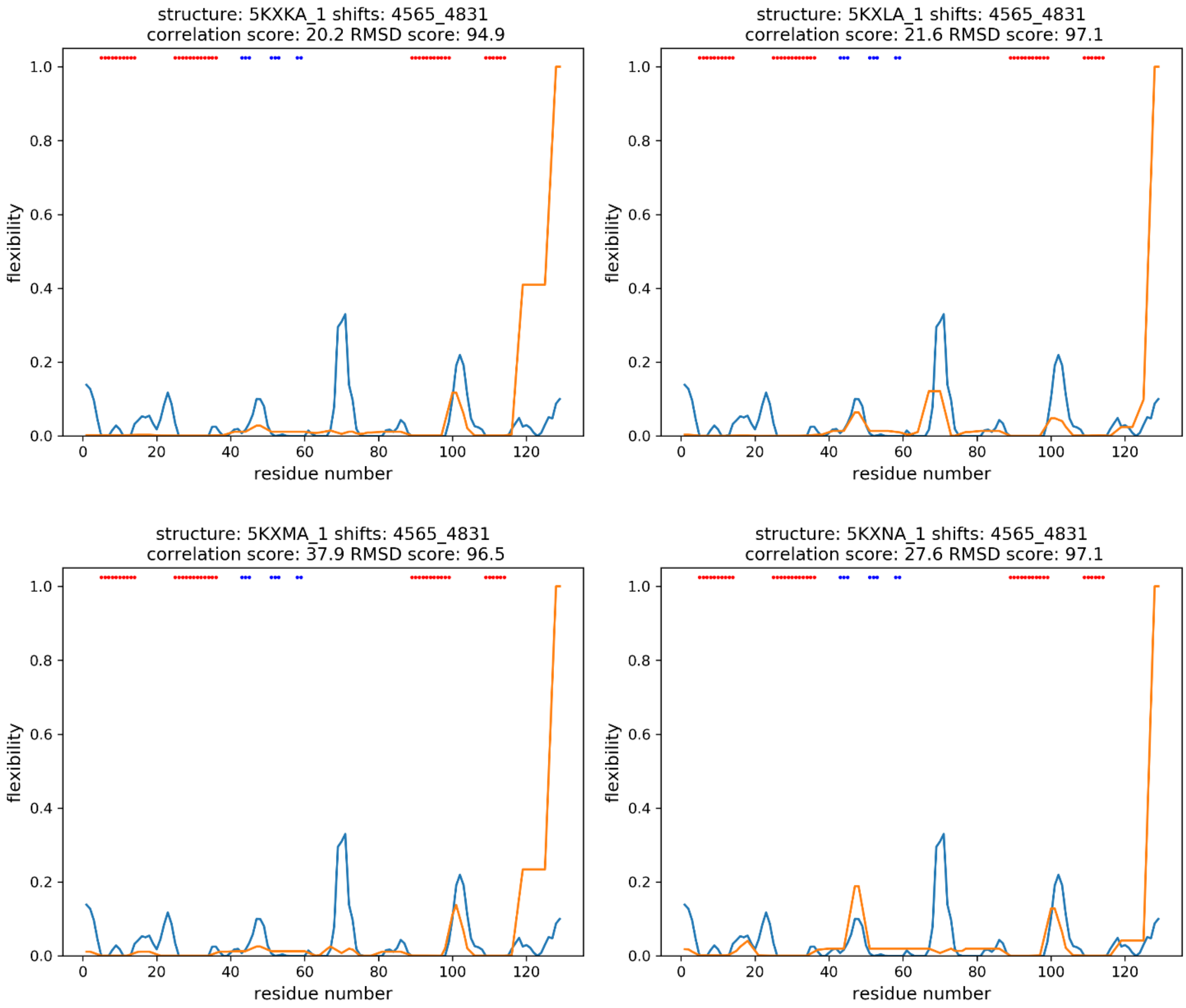

Supplementary Fig. 5. Comparison of flexibility predicted by RCI and FIRST for lysozyme X-ray structures obtained at 278 K. Continued on the next page.

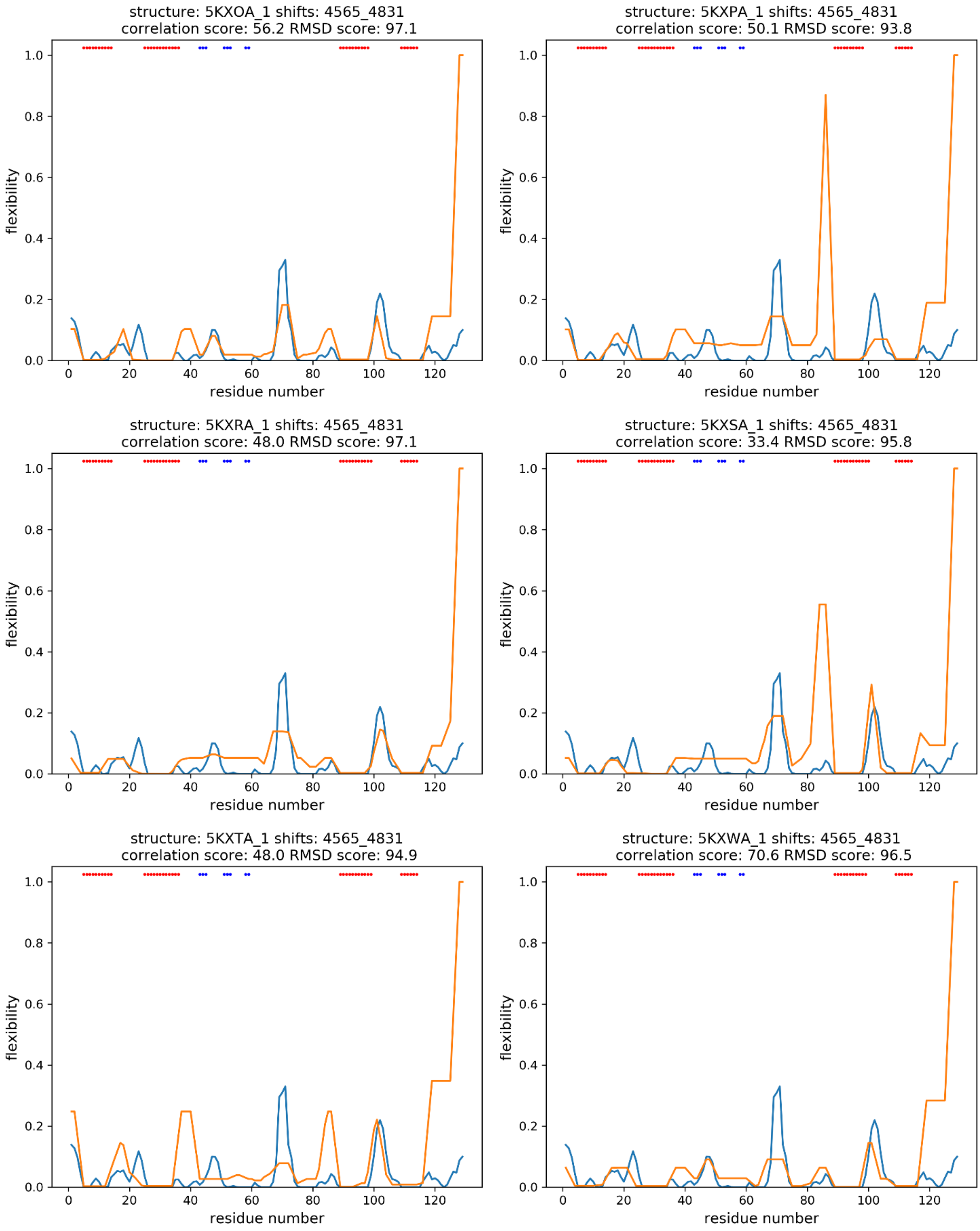

Supplementary Fig. 5 continued. Comparison of flexibility predicted by RCI and FIRST for lysozyme X-ray structures obtained at 278 K.

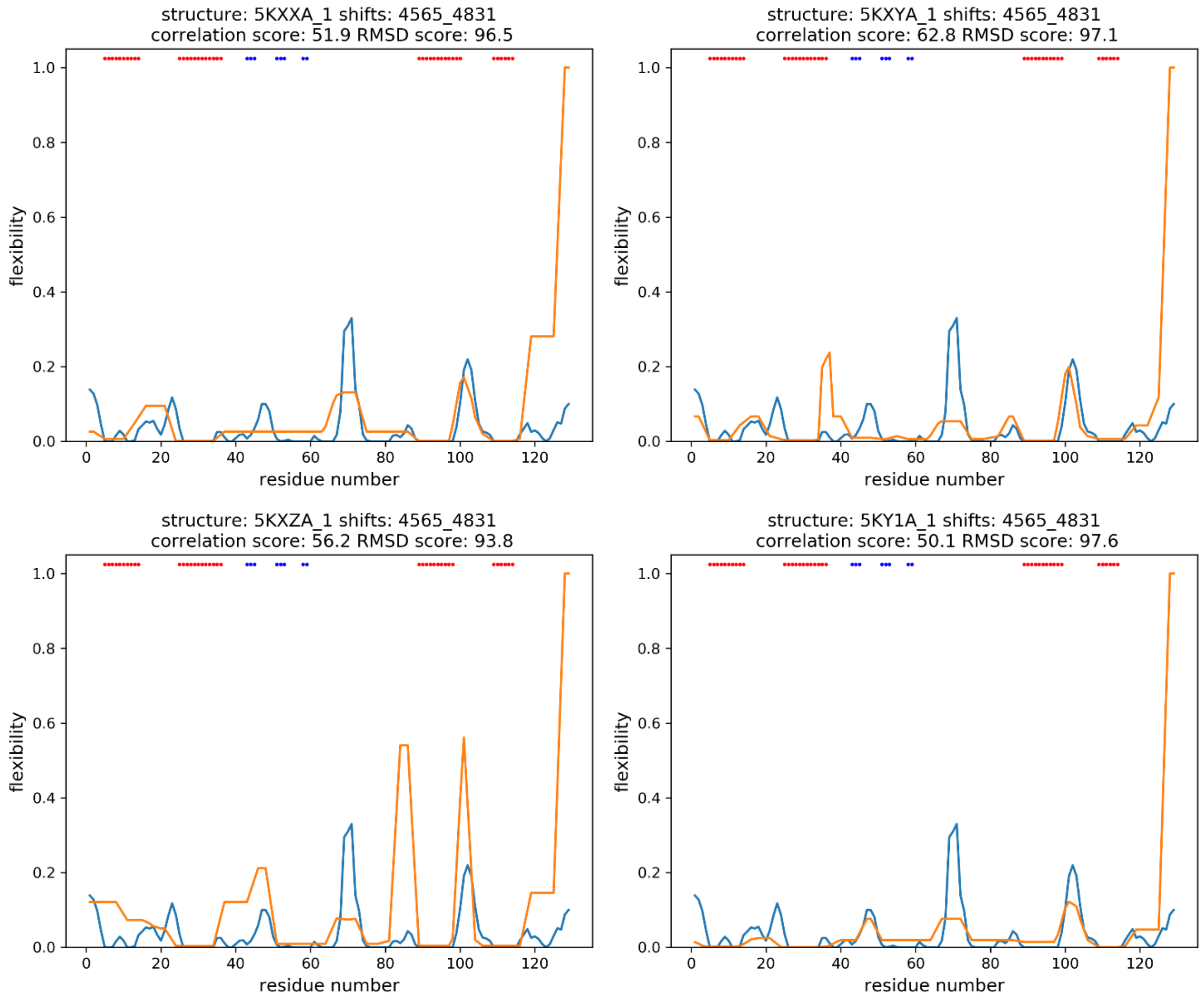

Supplementary Fig. 6. Backbone superposition of lysozyme X-ray structures obtained at 100 K (blue) and 278 K (orange).

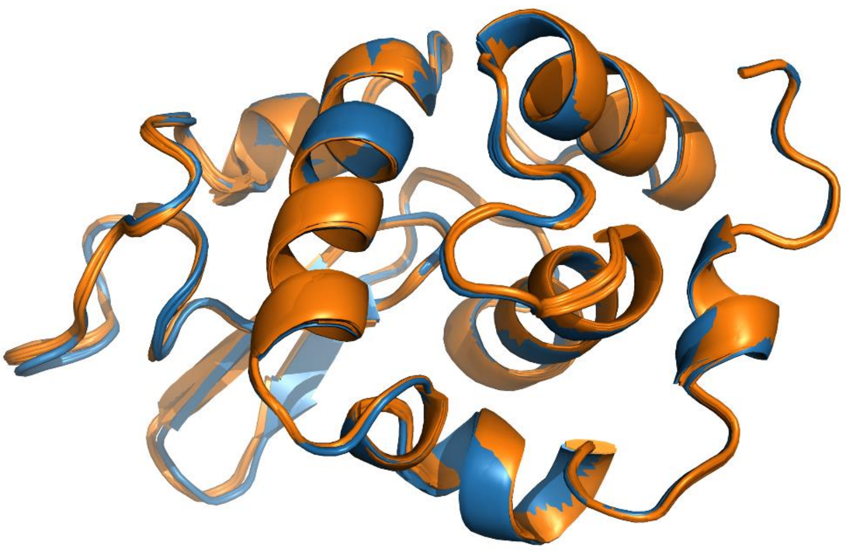

Supplementary Figure 7. There is a relationship between chemical shift completeness and ANSURR scores due to the relationship between chemical shift completeness and the accuracy of RCI. Sets of chemical shifts with completeness ranging between 20 and 95%, in increments of 5%, were randomly generated from the corresponding BMRB shift files for 6 NMR structures. Each set of shifts was generated 20 times and used to validate the structures using ANSURR. The mean RMSD score and correlation score as a function of shift completeness are plotted for each structure. The error bars show the standard error of the mean (*n*=20). RMSD score tends to plateau once shift completeness hits about 40%, whereas correlation score only begins to plateau at around 75-80% completeness.

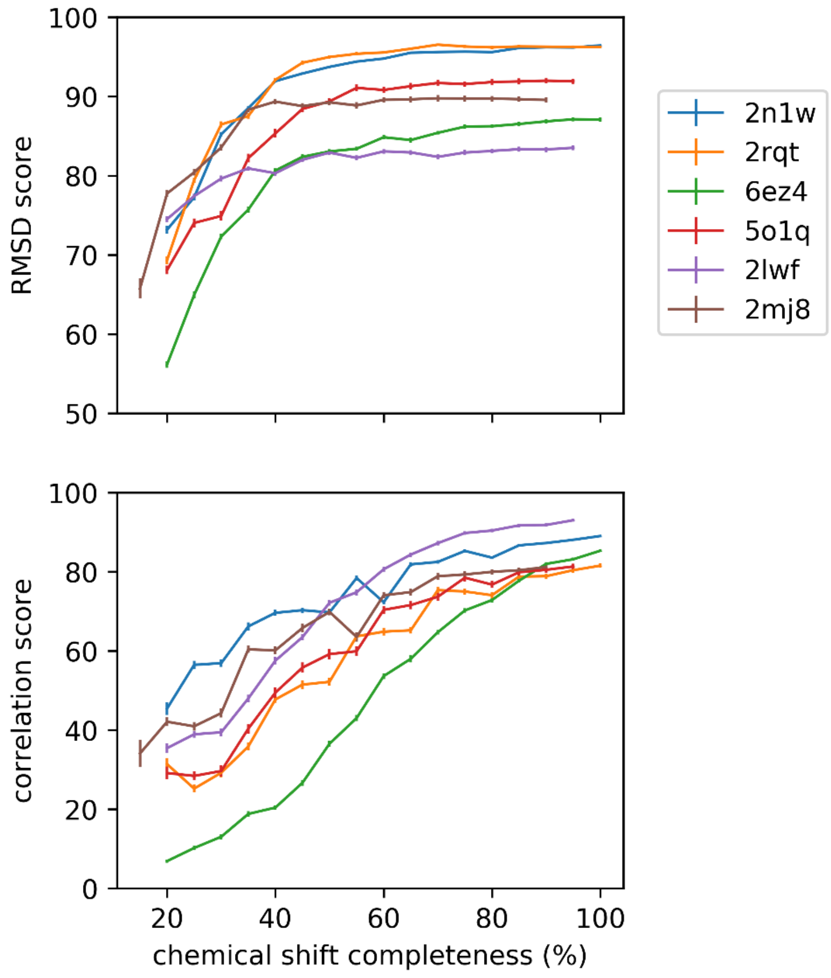

Supplementary Figure 8. Frequency distributions of output values for RCI (a) and FIRST (b). RCI values comprise residues from 7499 BMRB chemical shift entries related to NMR solution structures from the PDB. FIRST values comprise residues from 334 ensembles from the RECOORD CNW dataset[5]. Panel c compares these frequency distributions after rescaling RCI as outlined in the methods section of the manuscript. Overall, there is reasonable agreement between the frequency distributions. As we use the CNW dataset for much of our analyses, we decided to compare the frequency distribution of FIRST for a random selection of 408 NMR solution structures from the PDB (d). Here we also see reasonable agreement, although less good than for the CNW dataset. This is expected as structures in the CNW dataset are likely to be more accurate than a random structure from the PDB.

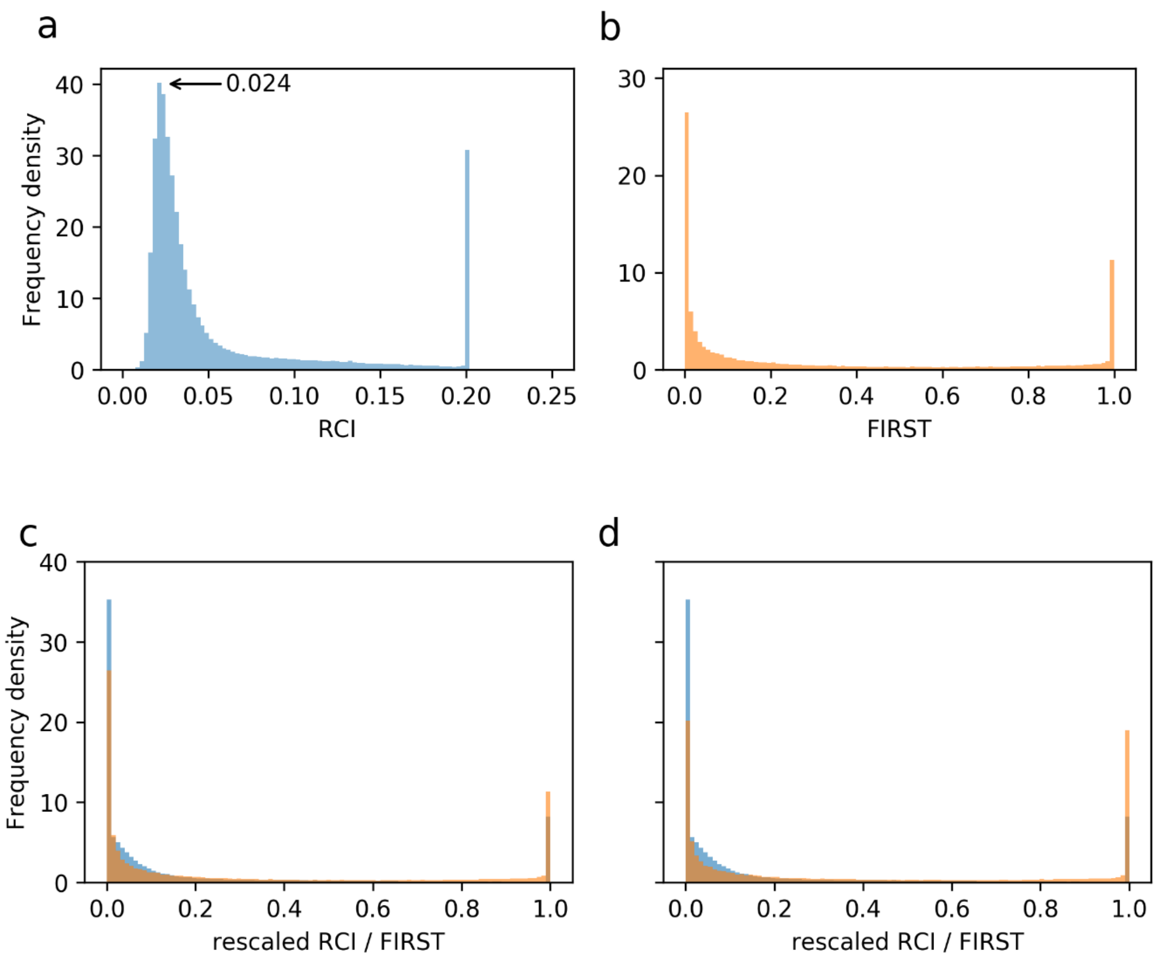

Supplementary Table 3. BMRB and PDB IDs for a set of comparable X-ray and NMR structures.

BMRB ID	X-ray PDB ID	NMR PDB ID
4019	1PLC	1TKW
4031	1RUV,3RN3	2AAS
4039	1RSY	1BYN,2K45,2K4A,2K8M
4052	1SNC	1JOK,1JOO,1JOQ,1JOR
4064	1HFC	1AYK,2AYK,3AYK,4AYK
4082	1FIL	1PFL
4094	2B8X	1BBN,1BCN,1ITI
4115	1EMV	1E0H,1IMP,1IMQ,2K5X
4162	1EPF	2NCM
4186	1CBS	1BLR
4198	1EZ3	1BR0
4202	1HL5	1BA9,1KMG,1RK7
4259	1RGE,1UCK	1C54
4296	1MJC	2L15
4317	1AIL	1NS1
4340	1JV4,1YP7	1DF3
4342	1EKG	1LY7
4354	1LAX,1FQA	1EZO,1EZP,2H25,2KLF
4371	1ONC	1PU3
4378	1G8I	2LCP
4401	1TOP,1NCX	1BLQ,1SKT,1TNP,1TNQ,<br>1ZAC
4421	1GXQ	1QQI
4425	1BDO	3BDO
4438	1C44	1QND
4472	1U8T	1DJM
4553	1XUO	1DGQ
4566	1AYF	1L6U,1L6V
4717	1F46,1Y2G	1F7W,1F7X
4797	1IAZ	1KD6
4840	2CDN	1P4S
4857	2A0B	1FR0
4964	1A2P,1BRI	1BNR,1FW7
5058	1GNU	1KOT
5081	1B2V	1YBJ
5142	1IWT,1LZ1	1IY3,1IY4
5194	1TJM	1K5W
5206	1MHO	1UWO
5211	1D4T	1KA7
5220	1I1J	1K0X
5275	1KQR	1KRI
5299	2VFX	1KLQ
5387	1UBQ	1D3Z,1G6J
5471	1P7T	1Y8B
5485	1W41,1H7M	1GO0
5712	1IPB	2GPQ
5756	1N0S	1T0V
5792	1EW4	1SOY

BMRB ID	X-ray PDB ID	NMR PDB ID
5843	1Q4R	1Q53
5898	1UOH	1TR4
5921	1VC1	1SBO
6075	1JL3	1Z2D,1Z2E
6122	1SMX,1SN8	1SLJ
6231	1UV0	2GO0
6375	1U07	1XX3
6503	1F2F	2JYQ
6504	1CNR	1CCM,1CCN,1YV8,1YVA,<br>2EYA,2EYB,2EYC,2EYD
6541	1CLL,1MXE,2F3Y	1CFC,1CFD,1CFF,1CKK,<br>1MUX,1NWD,1SY9,1X02,<br>2JZI,2K0E,2K0F,2KDU,<br>2KNE,2L53
6699	4ICB	1N65,2MAZ
6754	1QAV	1Z86
6776	1UJ8	2BZT
4070	1ZE3	1BF8
6090	1FF3	2GT3
6876	2NNR	2FO8
6922	2D3D	2FE9
6923	2AWG	2MF9
6932	1KBL	2FM4
6980	2D58	2G2B
15084	1TW4	1MVG,1ZRY,2JN3,2K62

Supplementary References

1. Berjanskii, M., Zhou, J., Liang, Y., Lin, G. & Wishart, D.S. Resolution-by-proxy: a simple measure for assessing and comparing the overall quality of NMR protein structures. *J Biomol NMR* **53**, 167-180 (2012).
2. Berjanskii, M. et al. PROSESS: a protein structure evaluation suite and server. *Nucleic Acids Research* **38**, W633-W640 (2010).
3. Deng, H., Jia, Y. & Zhang, Y. 3DRobot: automated generation of diverse and well-packed protein structure decoys. *Bioinformatics* **32**, 378-387 (2016).
4. Zemla, A. LGA: a method for finding 3D similarities in protein structures. *Nucleic Acids Research* **31**, 3370-3374 (2003).
5. Nederveen, A.J. et al. RECOORD: A recalculated coordinate database of 500+ proteins from the PDB using restraints from the BioMagResBank. *Proteins: Struct. Funct. Bioinf.* **59**, 662-672 (2005).
