# Supplementary material for: A method for validating the accuracy of NMR protein structures
Source: Nat Commun. 2020 Dec 18;11:6321. doi: 10.1038/s41467-020-20177-1 (PMC7749147; doi:10.1038/s41467-020-20177-1)
Supplement: Supplementary file 3 — Reporting Summary [file 41467_2020_20177_MOESM3_ESM.pdf]

nature research

Corresponding author(s): M P Williamsson and A Sljoka

Last updated by author(s): Oct 29, 2020

Reporting Summary

Nature Research wishes to improve the reproducibility of the work that we publish. This form provides structure for consistency and transparency in reporting. For further information on Nature Research policies, see our Editorial Policies and the Editorial Policy Checklist.

Statistics

For all statistical analyses, confirm that the following items are present in the figure legend, table legend, main text, or Methods section.

n/a	Confirmed	
☐	☒	The exact sample size (n) for each experimental group/condition, given as a discrete number and unit of measurement
☒	☐	A statement on whether measurements were taken from distinct samples or whether the same sample was measured repeatedly
☐	☒	The statistical test(s) used AND whether they are one- or two-sided<br>*Only common tests should be described solely by name; describe more complex techniques in the Methods section.*
☒	☐	A description of all covariates tested
☒	☐	A description of any assumptions or corrections, such as tests of normality and adjustment for multiple comparisons
☐	☒	A full description of the statistical parameters including central tendency (e.g. means) or other basic estimates (e.g. regression coefficient) AND variation (e.g. standard deviation) or associated estimates of uncertainty (e.g. confidence intervals)
☐	☒	For null hypothesis testing, the test statistic (e.g. F, t, r) with confidence intervals, effect sizes, degrees of freedom and P value noted<br>*Give P values as exact values whenever suitable.*
☒	☐	For Bayesian analysis, information on the choice of priors and Markov chain Monte Carlo settings
☒	☐	For hierarchical and complex designs, identification of the appropriate level for tests and full reporting of outcomes
☐	☒	Estimates of effect sizes (e.g. Cohen's d, Pearson's r), indicating how they were calculated

Our web collection on statistics for biologists contains articles on many of the points above.

Software and code

Policy information about availability of computer code

Data collection	RCI code was written locally (and is contained within the Github release detailed below) and was based on the code publicly available from http://wishart.biology.ualberta.ca/download/rci (version rci_v_1n_10_6_12_A.py). Random coil weighting coefficients were extracted from this code. FIRST code was adapted from a development version given to the authors by the original authors of FIRST. It has been modified to disable most of the functions of FIRST, limiting these to the rigid cluster decomposition calculation. The modified FIRST code is provided as an executable contained within the GitHub release. Output from the 3DRobot web server (https://zhanglab.ccmb.med.umich.edu/3DRobot), Molprobity (version 4.5), ResProx web server (http://www.resprox.ca) and PROSESS web server (https://www.prosess.ca) was used without modification. MODELLER version 9.17 and REDUCE version 3.23 were used.
Data analysis	Code is available at GitHub.com/nickjf/ANSURR, DOI 10.5281/zenodo.4161586

For manuscripts utilizing custom algorithms or software that are central to the research but not yet described in published literature, software must be made available to editors and reviewers. We strongly encourage code deposition in a community repository (e.g. GitHub). See the Nature Research guidelines for submitting code & software for further information.

Data

Policy information about availability of data

All manuscripts must include a data availability statement. This statement should provide the following information, where applicable:

- Accession codes, unique identifiers, or web links for publicly available datasets
- A list of figures that have associated raw data
- A description of any restrictions on data availability

Source data are listed in Supplementary Information and are from publicly available databases: specifically, the Protein Data Bank (www.rcsb/org), Biological Magnetic Resonance Bank (BMRB: www.bmrb.io) and RECOORD (www.ebi.ac.uk/pdbe/recalculated-nmr-data). The accession codes of PDB and BMRB entries used

April 2020

in this study are listed in the Supplementary Information file. Data supporting the findings of this work are available within the paper and its Supplementary Information. The datasets generated and analysed during the current study are available from the corresponding author (MPW) upon request.

Field-specific reporting

Please select the one below that is the best fit for your research. If you are not sure, read the appropriate sections before making your selection.

- [x] Life sciences
- [ ] Behavioural & social sciences
- [ ] Ecological, evolutionary & environmental sciences

For a reference copy of the document with all sections, see nature.com/documents/nr-reporting-summary-flat.pdf

Life sciences study design

All studies must disclose on these points even when the disclosure is negative.

Sample size	All structures in the RECOORD database were used. 300 decoy calculations were carried out on 79 of the ensembles in the RECOORD CNW75 dataset. We made no attempt to generate statistical measures from these, so the number of structures was determined by the observation that no new or different results were appearing on the addition of new structures.
Data exclusions	Data exclusions relate to the completeness and quality of data and are described in the paper.
Replication	The algorithm was tested and refined repeatedly. However once we had produced a reliable program, we stuck to the same program throughout to ensure comparability of results. Repeat calculations gave identical results.
Randomization	All structures in the RECOORD database with at least 75% chemical shift completeness were used. To generate decoys, we selected 79 ensembles from the CNW75 dataset that scored reasonably well according to ANSURR and represented a range of different fold types.
Blinding	Blinding is not relevant, because we were not carrying out statistical comparisons between groups.

Reporting for specific materials, systems and methods

We require information from authors about some types of materials, experimental systems and methods used in many studies. Here, indicate whether each material, system or method listed is relevant to your study. If you are not sure if a list item applies to your research, read the appropriate section before selecting a response.

Materials & experimental systems

n/a	Involved in the study	
x		Antibodies
x		Eukaryotic cell lines
x		Palaeontology and archaeology
x		Animals and other organisms
x		Human research participants
x		Clinical data
x		Dual use research of concern

Methods

n/a	Involved in the study	
x		ChIP-seq
x		Flow cytometry
x		MRI-based neuroimaging
