# Supplementary material for: A method for validating the accuracy of NMR protein structures
Source: Nat Commun. 2020 Dec 18;11:6321. doi: 10.1038/s41467-020-20177-1 (PMC7749147; doi:10.1038/s41467-020-20177-1)
Supplement: Supplementary file 2 — Peer Review File [file 41467_2020_20177_MOESM2_ESM.pdf]

Editorial Note: This manuscript has been previously reviewed at another journal that is not operating a transparent peer review scheme. The manuscript was considered suitable for publication without further review at *Nature Communications*.
